# Structural insights into ligand recognition and selectivity of the human hydroxycarboxylic acid receptor HCAR2

**DOI:** 10.1038/s41421-023-00610-7

**Published:** 2023-11-28

**Authors:** Xin Pan, Fang Ye, Peiruo Ning, Zhiyi Zhang, Xinyu Li, Binghao Zhang, Qian Wang, Geng Chen, Wei Gao, Chen Qiu, Zhangsong Wu, Jiancheng Li, Lizhe Zhu, Jiang Xia, Kaizheng Gong, Yang Du

**Affiliations:** 1 Kobilka Institute of Innovative Drug Discovery, Shenzhen Futian Biomedical Innovation R&D Center, School of Medicine, The Chinese University of Hong Kong, Shenzhen, Guangdong China; 2 Department of Cardiology, Central Laboratory, The Affiliated Hospital of Yangzhou University, Yangzhou University, Yangzhou, Jiangsu China; 3 Warshel Institute for Computational Biology, School of Medicine, The Chinese University of Hong Kong, Shenzhen, Guangdong China; 4 Instrumental Analysis Center, Shenzhen University, Shenzhen, Guangdong China; 5 Department of Chemistry, The Chinese University of Hong Kong, Shatin, Hong Kong SAR, China

**Keywords:** Cryoelectron microscopy, Cell signalling

## Abstract

Hydroxycarboxylic acid receptor 2 (HCAR2) belongs to the family of class A G protein-coupled receptors with key roles in regulating lipolysis and free fatty acid formation in humans. It is deeply involved in many pathophysiological processes and serves as an attractive target for the treatment of cardiovascular, neoplastic, autoimmune, neurodegenerative, inflammatory, and metabolic diseases. Here, we report four cryo-EM structures of human HCAR2–Gi1 complexes with or without agonists, including the drugs niacin (2.69 Å) and acipimox (3.23 Å), the highly subtype-specific agonist MK-6892 (3.25 Å), and apo form (3.28 Å). Combined with molecular dynamics simulation and functional analysis, we have revealed the recognition mechanism of HCAR2 for different agonists and summarized the general pharmacophore features of HCAR2 agonists, which are based on three key residues R111^3.36^, S179^45.52^, and Y284^7.43^. Notably, the MK-6892–HCAR2 structure shows an extended binding pocket relative to other agonist-bound HCAR2 complexes. In addition, the key residues that determine the ligand selectivity between the HCAR2 and HCAR3 are also illuminated. Our findings provide structural insights into the ligand recognition, selectivity, activation, and G protein coupling mechanism of HCAR2, which shed light on the design of new HCAR2-targeting drugs for greater efficacy, higher selectivity, and fewer or no side effects.

## Introduction

Hydroxycarboxylic acid receptor 2 (HCAR2), also known as GPR109A, is an important metabolite-sensing receptor present in most mammalian species, and belongs to the class A G protein-coupled receptor (GPCR) family^1–3^. HCAR2 is highly expressed in multiple cell types (e.g., adipocytes, vascular endothelium, immune cells, retinal pigmented cells, colonic epithelial cells, keratinocytes, and microglia) and mediates downstream signaling transducers by coupling to the Gi/o family of G proteins^4,5^. The endogenous ligands of HCAR2 are β-hydroxybutyrate (β-OHB) and butyrate, both of which serve as nutrient sources for cells under various physiological conditions^2,6^. In particular, upon starvation or other extreme conditions, HCAR2 is activated by elevated β-OHB in vivo to reduce the lipolysis and free fatty acid formation in adipocytes, thus promoting efficient utilization of fat energy stores and preventing the development of ketoacidosis^7,8^. Moreover, HCAR2 is implicated in mitigating many pathophysiological processes, including the reduction of chemokine and pro-inflammatory cytokine production; amelioration of atherosclerosis, sepsis, and diabetic retinopathy; suppression of the occurrence of breast cancer, colitis, and acute pancreatitis; and maintenance of the integrity of the intestinal barrier^5,9,10^. Emerging studies also indicate that the activation of microglial HCAR2 has beneficial effects on multiple neurological disorders, such as Alzheimer’s disease, Parkinson’s disease, multiple sclerosis, stroke, and pathological pain conditions^11,12^. Collectively, HCAR2 is emerging as an attractive therapeutic target for the treatment of cardiovascular, neoplastic, autoimmune, neurodegenerative, inflammatory, and metabolic diseases.

Currently, several highly potent HCAR2 agonists, including niacin, acipimox, acifran, and monomethyl fumarate (MMF), have been approved for clinical treatment of cardiovascular and neurological disorders^13^. Of these, niacin serves as a well-known agonist of HCAR2. It is the oldest lipid-lowering drug to date, resulting from its ability to lower very low-density lipoprotein cholesterol, low-density lipoprotein cholesterol, and lipoprotein levels, as well as to increase high-density lipoprotein cholesterol levels to a greater extent than many other marketed drugs^14^. In addition, niacin has well-established antiatherogenic effects which, to a considerable degree, are based on the HCAR2-mediated anti-inflammatory effects of reducing M1 macrophage proportion^15,16^. The latest research also showed that niacin is being investigated as a drug for Parkinson’s disease and glioblastoma because of its immunomodulatory and neuroprotective properties, and clinical trials are currently in progress (NCT03808961 and NCT04677049)^17^. The niacin-derived anti-lipolytic drugs acipimox and acifran are generally used to treat dyslipidemia and atherosclerosis clinically^18^. Moreover, MMF was approved by the FDA in 2020 for the treatment of relapsing multiple sclerosis^11,19^. It has been demonstrated that HCAR2 at least in part mediates the beneficial effects of MMF. This was confirmed by a study by Parodi et al., in which MMF was shown to switch the LPS-activated microglia from a pro-inflammatory type to a neuroprotective type through activation of HCAR2^20^. Overall, the agents targeting HCAR2 have achieved notable successes in treating a variety of clinical diseases; nevertheless, several important challenges still remain. First, despite the good treatment efficacy of niacin, acipimox, and acifran, their use is less widespread than statins for the treatment of lipid disorders, which is mainly attributed to an uncomfortable cutaneous flushing effect that limits patient compliance^21^. Given this, some highly subtype-specific HCAR2 agonists (e.g., MK-6892, SCH900271, and GSK256073) have been developed, which share the lipid-lowering effects, but significantly alleviate the flushing effect^22–24^. This leads us to question what the structural differences between these subtype-specific agonists and approved drugs are when bound to HCAR2. Recently, several experimental structures of HCAR2 bound to ligands have been reported successively, but the detailed binding modes and recognition mechanisms of endogenous ligands, therapeutic agents, and subtype-specific HCAR2 agonists have not been systematically explored^25,26^. Second, the most homologous protein to HCAR2 is the same subfamily receptor HCAR3 (GPR109B), exclusively found in humans and higher primates such as chimpanzees^27^. Notably, HCAR2 shares up to 96% sequence identity with HCAR3, which to some extent increases the difficulty for drug development when selectively targeting the HCAR2 receptor^28^. A clear example is the niacin and acipimox, which target both HCAR2 and HCAR3, although with a much lower affinity to HCAR3 than to HCAR2^29^. Last, HCAR2 elicits its physiological responses by coupling primarily to Gi/o proteins to inhibit adenylate cyclase and cyclic AMP signaling. The activation and G protein coupling mechanisms underlying HCAR2 are still elusive.

In this study, we employed single-particle cryo-electron microscopy (cryo-EM) to determine the structures of human HCAR2 in complex with heterotrimeric Gi1 protein: HCAR2 bound to the drugs niacin and acipimox; HCAR2 bound to the highly subtype-specific agonist MK-6892; and HCAR2 in the absence of a ligand (apo) state. Combined with molecular simulation and mutagenesis results, our study provides a structural framework for understanding the ligand recognition and selectivity, receptor activation, and G protein coupling mechanism of HCAR2. More importantly, we believe that these accurate structure templates will accelerate the development of HCAR2-targeting drugs with greater efficacy, higher selectivity, and fewer or no side effects.

## Results

### The overall structure of the HCAR2–Gi complex

**Fig. 1:**
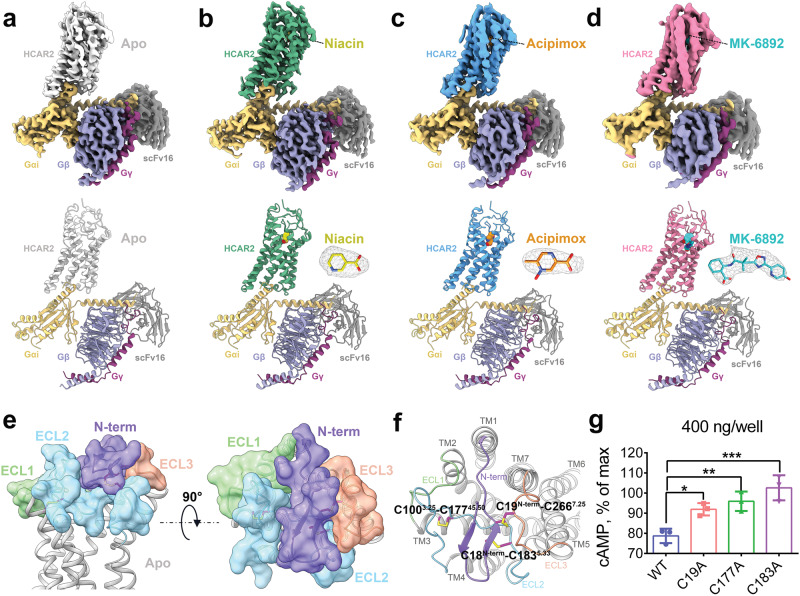
Cryo-EM structures of HCAR2–Gi1 in the apo and niacin-, acipimox-, MK-6892-bound forms. **a**–**d** Cryo-EM maps and structural models of HCAR2–Gi1 signaling complex in the absence (**a**) or presence of niacin (**b**), acipimox (**c**), and MK-6892 (**d**). The densities of the agonists (shown as sticks) are depicted as gray meshes. The maps and structural models are colored by subunits. Light gray, apo-HCAR2; forest green, niacin–HCAR2; deep sky blue, acipimox–HCAR2; hot pink, MK-6892–HCAR2; light yellow, Gαi; slate blue, Gβ; dark magenta, Gγ; dark gray, scFv16; yellow, niacin; dark orange, acipimox; cyan, MK-6892. **e** Extracellular architecture of apo-HCAR2 from side and top views. The N-terminal loop (blue purple), ECL1 (light green), ECL2 (sky blue), and ECL3 (coral) are shown as transparent surface presentations and overlaid on the cartoon model of HCAR2 (light gray). **f** Three disulfide bonds (magenta sticks) are formed in the extracellular region of HCAR2. The N-terminal loop (blue purple), ECL1 (light green), ECL2 (sky blue), and ECL3 (coral) are shown as cartoon models. **g** Effects on Gi-mediated cAMP by single-point mutations of C19^N-term^, C177^45.50^, and C183^5.33^ that disrupt the disulfide bonds. The data are presented as mean ± SEM, one-way analysis of variance (ANOVA), *P < 0.05, **P < 0.01, ***P < 0.001. The experiments were performed in triplicate.

To investigate the molecular mechanisms of HCAR2 in ligand recognition and signal transduction, we prepared a stable HCAR2–Gi1 complex through co-expression of three subunits of the Gi1 protein and HCAR2 receptor in Sf9 insect cells. Immediately afterward, the HCAR2–Gi1 complex was assembled with scFv16, a Gi-stabilizing antibody, in the absence or presence of an agonist, thus obtaining the cryo-EM density maps of four different complexes with overall resolutions of 3.28 Å (apo), 2.69 Å (niacin), 3.23 Å (acipimox), and 3.25 Å (MK-6892) (Fig. 1a–d). The majority of the side chains of HCAR2 and the Gi1 protein residues were well defined in all obtained complexes, providing accurate models of intermolecular interactions of HCAR2 with the ligand and Gi1 (Supplementary Figs. S1–S4). It can clearly be seen that HCAR2 displayed the canonical GPCR topology of a heptahelical transmembrane bundle (7TM), connected by an extracellular N-terminus, three extracellular loops (ECL1–3) and three intracellular loops (ICL1–3). The plotted snake diagram of HCAR2 showed that it also contained an amphipathic helix VIII and a long C-terminus, although their electron densities were not observed in our cryo-EM maps, indicating highly flexible properties of these regions (Supplementary Fig. S5).

Despite binding to different ligands, the overall structures of the four active state HCAR2–Gi1 complexes resembled each other, with root-mean-square deviation values of 0.4–1.0 Å for the Cα atoms. A striking feature of HCAR2 was that the ECL2 region (K164–Q187) formed a lid that almost completely capped the extracellular vestibule (Fig. 1e). It seems that HCAR2 does not necessarily require ligand binding to activate the downstream signaling transducers, because its ECL2 may act as a built-in “agonist”.

To better understand the structural features of ECL2, sequence and structure alignment of HCAR2 with other reported GPCRs with self-activation, including GPR52, GPR17, and BILF1 were performed^30–32^. As shown in Supplementary Fig. S6a–d, although the overall sequence homology was quite low, all the ECL2 regions occupied the orthosteric binding site to different degrees. The only conserved residue in the ECL2 regions was Cys^45.50^, which formed a disulfide bond with Cys^3.25^ of TM3 in all four GPCRs (Fig. 1f and Supplementary Fig. S6e–g). Typically, this disulfide bond (Cys^45.50^–Cys^3.25^) between ECL2 and TM3 presents in most class A GPCRs and plays significant roles in maintaining the architecture of the ligand binding pocket as well as contributing to ligand recognition^33^. Additionally, HCAR2 exhibited several distinct features compared with these three GPCRs. The N-terminus C18^N-term^ and C19^N-term^ of HCAR2 formed two extra disulfide bonds with C183^5.33^ of ECL2 and C266^7.25^ of ECL3, respectively (Fig. 1f). By contrast, the N-terminus of GPR52 and BILF1 did not form a disulfide bond with ECL2, and only formed one with TM1 and ECL3, respectively (Supplementary Fig. S6e, g). Ultimately, under the interactions of a total of three disulfide bonds (C100^3.25^–C177^45.50^, C18^N-term^–C183^5.33^, C19^N-term^–C266^7.25^), HCAR2 displayed a unique extracellular architecture: the ECL2 was closely clamped by ECL1 and ECL3, as well as compressed by the N-terminus from the top (Fig. 1e).

Notably, the extracellular conformations of the agonist-bound HCAR2 complexes also appeared to be stabilized by three disulfide bonds (Supplementary Fig. S7b–d). In particular, we were able to observe the density maps of disulfide bonds clearly from the niacin–HCAR2 complex (Supplementary Fig. S7e). We then divided the ECL2 into three segments, which were named as P1 segment (K164–L176), P2 segment (C177–I182), and P3 segment (C183–Q187) (Supplementary Fig. S7a). Mutagenesis and cellular functional assays showed that replacing the ECL2 region, P2 segment, and P3 segment with a six-residue linker (GGSGGS), or mutating the residues C19^N-term^, C177^45.50^, and C183^5.33^ did not significantly affect HCAR2 expression (Supplementary Fig. S8), but all profoundly reduced the constitutive Gi-mediated cAMP signaling, due to the disruption of disulfide bonds (Fig. 1g and Supplementary Fig. S7f–h). Therefore, we considered that the multiple disulfide bonds formed further improved the structural stability of ECL2, which might be important for the activation of HCAR2.

### Ligand recognition of the HCAR2 receptor

**Fig. 2:**
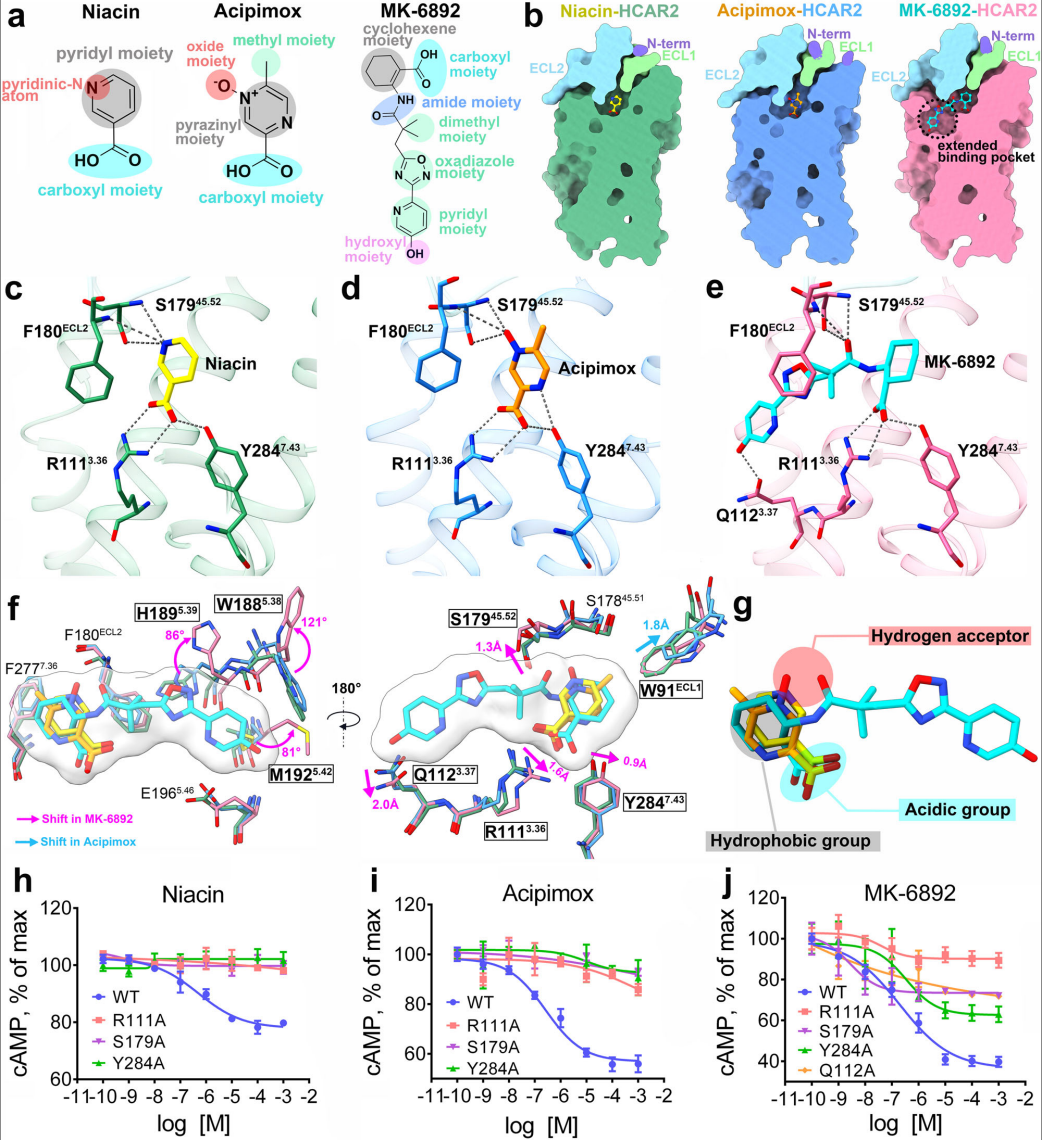
Ligand-binding pocket of active HCAR2 bound to different agonists. **a** Chemical structures of niacin, acipimox, and MK-6892. **b** Vertical cross-sections of the binding pockets of niacin, acipimox, and MK-6892 in HCAR2. **c**–**e** Detailed interactions of niacin, acipimox and MK-6892 with HCAR2. The polar interactions are indicated by dark gray dashed lines. **f** Superposition of the niacin, acipimox, and MK-6892 binding poses, as well as surrounding key residues. **g** General pharmacophore features common to most of the agonists recognized by HCAR2. The structures of HCAR2 and agonists are colored differently. Forest green, niacin–HCAR2; deep sky blue, acipimox–HCAR2; hot pink, MK-6892–HCAR2; blue purple, N-terminal loop; light green, ECL1; sky blue, ECL2; coral, ECL3; yellow, niacin; dark orange, acipimox; cyan, MK-6892. **h**–**j** Effects on Gi-mediated cAMP by single-point mutations of R111^3.36^, Y284^7.43^, S179^45.52^, and Q112^3.37^ that interact with niacin, acipimox, and MK-6892. The data are presented as mean ± SEM. The experiments were performed in triplicate.

Both niacin (EC_50_ = 0.06–0.25 μM) and acipimox (EC_50_ = 2.6–6 μM) are representative drugs targeting HCAR2, while MK-6892 (EC_50_ = 0.016 μM) is a highly subtype-specific agonist of HCAR2 with a higher affinity (Fig. 2a)^3,22,34^. The GTP turnover assay was used to further confirm the Gi1 activation by niacin, acipimox, and MK-6892 in vitro. The results suggested that niacin and acipimox could activate both HCAR2 and HCAR3 receptors, but preferred HCAR2 (Supplementary Fig. S9a, b). In contrast, the subtype-specific agonist MK-6892 barely activated HCAR3. Interestingly, we noted that the density maps for the agonists were a bit ambiguous, especially in the position of pyridinic-N atom of niacin and the oxide moiety of acipimox. It seemed that the pyridinic-N atom of niacin (or oxide moiety of acipimox) could turn either toward the S179^45.52^ or toward the Y87^2.64^ orientation, both of which fit the density maps (Supplementary Fig. S10a–d). To determine the correct binding poses of niacin and acipimox, we performed a detailed interaction analysis and found that the pyridinic-N atom of niacin (or oxide moiety of acipimox) could form much more hydrogen bonds with the S179^45.52^ and F180^ECL2^ than with the Y87^2.64^ (Supplementary Fig. S10a–d). Moreover, the molecular dynamics (MD) simulations were employed and the results suggested that both the ligands niacin and acipimox could not form stable hydrogen bonds and salt bridge when they were oriented in the Y87^2.64^ direction (Supplementary Fig. S11). However, in marked contrast, more stable binding poses were observed when the pyridinic-N atom of niacin and oxide moiety of acipimox were inclined to the S179^45.52^ direction, because of the longer lifetime of salt bridge and hydrogen bond networks (Supplementary Fig. S12). Consistently, mutation of S179^45.52^A obviously affected the agonistic activity for niacin and acipimox, while mutation of Y87^2.64^A did not (Fig. 2h, i and Supplementary Fig. S10f, g). Thus, the correct binding poses were toward the S179^45.52^ orientation.

When focusing on the structural details of niacin and acipimox complexes, we found that these two ligands adopted a very similar binding pose and were deeply embedded in the orthosteric pocket constituted by TM2, TM3, TM5, TM6, TM7, ECL1, and ECL2 (Fig. 2b and Supplementary Fig. S7b–d). In particular, ECL1 and ECL2 were located on the top of the pocket and almost completely isolated the ligands from the extracellular milieu. Further analysis revealed that niacin and acipimox bound to HCAR2 primarily through polar interactions (Fig. 2c, d). Specifically, the positively charged residue R111^3.36^ of HCAR2 was thought to be the most critical residue for binding niacin and acipimox by forming a strong salt bridge with a negatively charged carboxyl group of ligands. The cAMP accumulation assay suggested that the mutation of R111^3.36^ to alanine led to a significant loss of agonistic activity for niacin and acipimox (Fig. 2h, i). Furthermore, the interactions were also stabilized by four pairs of hydrogen bonds: the carboxyl moiety of niacin (or acipimox) with the side chain of Y284^7.43^; and the pyridinic-N atom of niacin (or the oxide moiety of acipimox) with the side chain and backbone of S179^45.52^, as well as with the backbone of F180^ECL2^ (Fig. 2c, d). Consistently, the mutation in either Y284^7.43^ or S179^45.52^ markedly reduced ligand activity (Fig. 2h, i). In addition, a subtle conformational difference existed between the orthosteric pockets of niacin and acipimox complexes. Since acipimox has an additional methyl moiety at the 5-position of the pyrazine ring, W91^ECL1^ was observed to move 1.8 Å toward the top of the pocket so as to accommodate this group (Fig. 2f).

The ligand-binding pocket formed by bulky MK-6892 was comprised of two subpockets: a canonical orthosteric binding pocket and an extended binding pocket (Fig. 2b). Similar to niacin and acipimox, the carboxyl, cyclohexene, and amide moieties of MK-6892 primarily occupied the orthosteric pocket and formed similar interactions with R111^3.36^, Y284^7.43^, S179^45.52^, and F180^ECL2^ (Fig. 2e and Supplementary Fig. S10e). A notable difference was that the top hydroxyl group of MK-6892 formed a hydrogen bond with Q112^3.37^. Furthermore, the dimethyl, oxadiazole, and pyridyl moieties of MK-6892 were mainly positioned on the extended binding pocket formed by TM3, TM4, and TM5. The mutations of R111^3.36^, Y284^7.43^, S179^45.52^, and Q112^3.37^ to alanine significantly impaired the MK-6892 activity, further confirming their important roles in MK-6892 binding (Fig. 2j). Following the above analysis, we speculated that the extra interaction, particularly mediated by Q112^3.37^ in the extended binding pocket, contributed to the high binding affinity of MK-6892. Of course, to accommodate such interaction patterns, several minor changes in chemical shifts were observed in the key residues of the MK-6892 complex. For example, compared with the niacin and acipimox complexes, the side chains of R111^3.36^, Q112^3.37^, S179^45.52^, and Y284^7.43^ were found to move 1.6, 2.0, 1.3, and 0.9 Å, respectively, to better make polar interactions with the bulky MK-6892 (Fig. 2f). But more importantly, how did the extended binding pocket form in the MK-6892-HCAR2 complex? We noted that the oxadiazole and pyridyl groups of MK-6892 forced the side chains of H189^5.39^ and M192^5.42^ to rotate about 86° and 81°, respectively, thereby avoiding to clash with the ligand (Fig. 2f). Immediately afterward, the rotation of M192^5.42^ occupied the position initially occupied by W188^5.38^ and caused it to rotate upward about 121°. Eventually, these large conformational changes of W188^5.38^, H189^5.39^, and M192^5.42^ in the side chain orientations together led to the extended binding pocket formation.

It was still of note that the featured aromatic rings of niacin, acipimox and MK-6892 were surrounded by a series of hydrophobic residues in the orthosteric pocket, including L83^2.60^, W91^ECL1^, L104^3.29^, L107^3.32^, F180^ECL2^, F277^7.36^, and L280^7.39^ (Supplementary Fig. S13a, b). The alanine substitutions of these residues influenced the agonistic activity to varying degrees (Supplementary Fig. S13c–e). Thus, the hydrophobic environment in the orthosteric pocket was necessary for HCAR2 activation as well. Overall, the hydrophilic, hydrophobic, and charged properties of niacin, acipimox and MK-6892 matched quite well with those of the orthosteric pocket: the upper portion of the binding site (toward the extracellular side) was largely hydrophobic, while the bottom portion (intracellular side) was hydrophilic and charged (Supplementary Fig. S14).

### Pharmacophore features of the HCAR2 agonist

In the previous section, we had provided important insights into the key roles played by R111^3.36^, Q112^3.37^, S179^45.52^, and Y284^7.43^ in ligand recognition of HCAR2. To further prove this, surface plasmon resonance (SPR) was performed to measure the affinity between the agonists and purified wild-type HCAR2 as well as its mutants (Supplementary Fig. S15a–f). The SPR assay revealed that HCAR2 displayed the highest binding affinity to MK-6892 (*K*_D_ = 0.022 μM), followed by niacin (*K*_D_ = 0.058 μM), and lowest with acipimox (*K*_D_ = 0.429 μM) (Supplementary Fig. S15a, f). Mutations of S179^45.52^A and Y284^7.43^A markedly reduced the affinity of HCAR2 to all three agonists. Besides, mutation of Q112^3.37^A had a negative effect on the affinity of HCAR2 to MK-6892, indicating the important role of this site for MK-6892 binding (Supplementary Fig. S15a, e). Most notably, the alanine replacement of R111^3.36^ almost completely abolished the binding of HCAR2 to all three agonists. Given this, we considered that the negatively charged acidic group of R111^3.36^ was indeed the most important and essential factor for the agonist-mediated HCAR2 activation. Consistently, previous studies have suggested that if the carboxyl group of niacin was replaced with an amide group, the produced nicotinamide was no longer active toward HCAR2^35^.

In order to reveal the structural features of ligand recognition of HCAR2, the chemical structures of all three agonists were analyzed. Specifically, niacin and acipimox share similar features, including a carboxyl moiety, an aromatic ring (pyridyl moiety of niacin and pyrazinyl moiety of acipimox), and an electron-rich moiety (pyridinic-N atom of niacin and oxide moiety of acipimox) (Fig. 2a). In comparison, MK-6892 is much more complicated. Structural analysis suggested that MK-6892 not only exhibited the shared three structural features of niacin and acipimox, but also had several unique groups, including dimethyl, oxadiazole, pyridyl, and hydroxy moieties (Fig. 2a).

On the basis of the acipimox-bound HCAR2 structure, we investigated the interactions of HCAR2 with more HCAR2 agonists, including endogenous ligands (butyrate and β-OHB), important drugs (MMF and acifran), and 3-pyridineacetic acid, through molecular docking (Supplementary Fig. S16a)^36^. The docking pose of acipimox reproduced the cryo-EM pose well, increasing the accuracy and reliability of the docking results (Supplementary Fig. S16b). The predicted binding modes suggested that all agonists were bound nicely in the orthosteric pocket. Except for butyrate, all other agonists mainly formed polar interactions with R111^3.36^, Y284^7.43^, and S179^45.52^, which were consistent with those of niacin and acipimox (Supplementary Fig. S16c–g). It is well known that both butyrate and β-OHB are endogenous ligands of HCAR2, but butyrate lacks one hydroxyl group in its structure, making it unable to form a hydrogen bond with S179^45.52^. This result provided a good explanation for why β-OHB has a higher potency for HCAR2 than butyrate^2,6^. Additionally, there were still hydrophobic interactions between these agonists and surrounding hydrophobic residues. The difference was that acifran and 3-pyridineacetic acid interacted mainly through a rigid aromatic ring, like that of niacin, acipimox, and MK-6892, whereas butyrate, β-OHB, and MMF interacted primarily via aliphatic chains. Combining the cryo-EM structures and docking results, we summarized the general pharmacophore features that might be common to most of the agonists recognized by HCAR2: an acidic group (contributes to the salt bridge and hydrogen bond with basic R111^3.36^ and Y284^7.43^), a hydrogen acceptor (contributes to the hydrogen bond with S179^45.52^), and a hydrophobic aliphatic or aromatic group (contributes to the hydrophobic interactions) (Fig. 2g).

### Ligand selectivity between HCAR2 and HCAR3 receptors

**Fig. 3:**
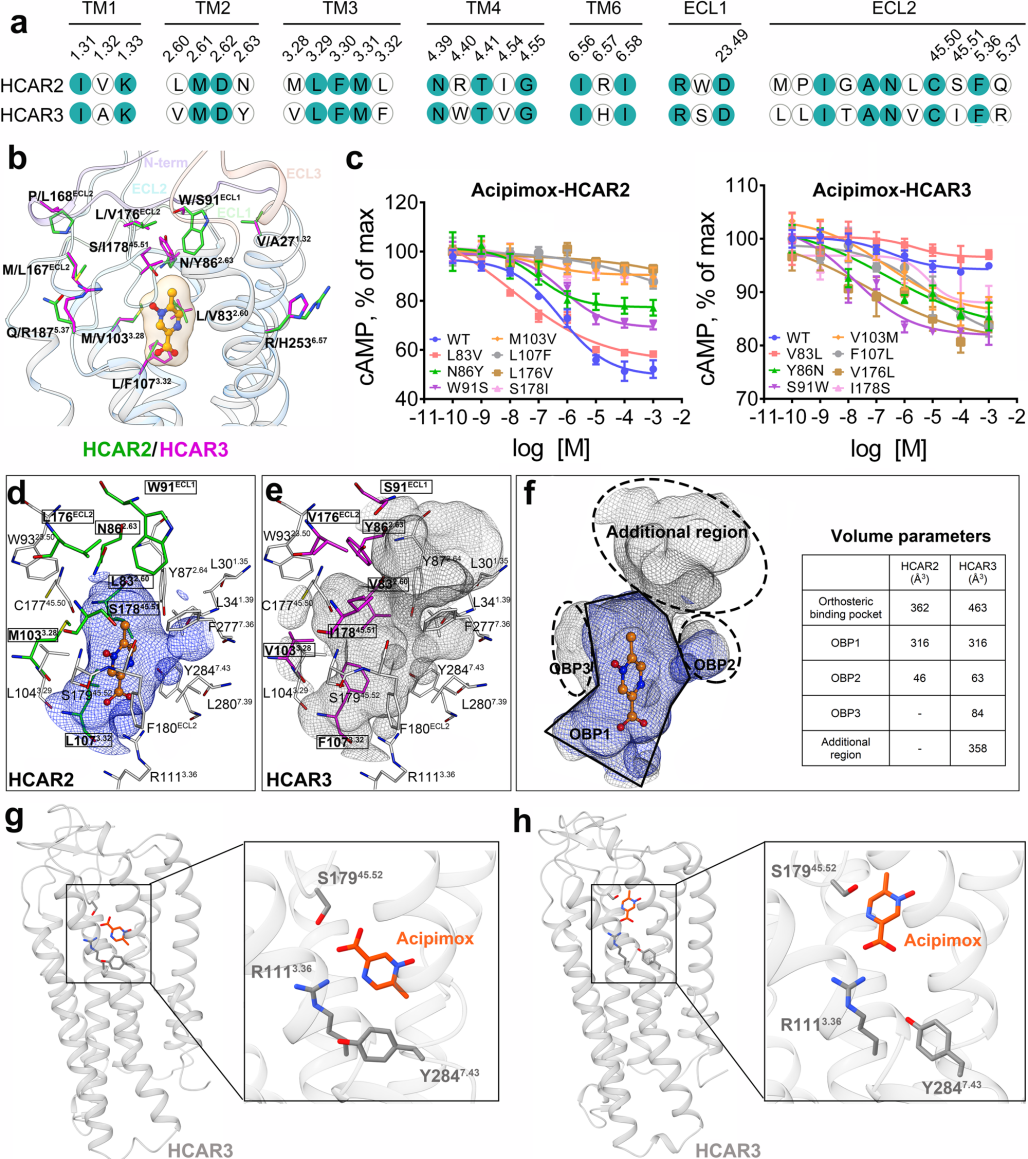
Structural basis for ligand selectivity between HCAR2 and HCAR3. **a** Sequence alignment of residues in HCAR2 and HCAR3. The conserved residues are highlighted in solid dark green circles. **b** Superposition of the 15 different residues. The N-terminal loop (blue purple), ECL1 (light green), ECL2 (sky blue), and ECL3 (coral) in HCAR2 (deep sky blue) and HCAR3 (gray) are overlaid in cartoon representation. The 15 different residues in the HCAR2 (green), HCAR3 (magenta), and acipimox (dark orange) are shown in stick representation. **c** Effects on Gi-mediated cAMP by single-point mutations at positions 83, 86, 91, 103, 107, 176, and 178 in HCAR2 and HCAR3, respectively. The data are presented as mean ± SEM. The experiments were performed in triplicate. **d**–**f** Orthosteric binding pockets of HCAR2 and HCAR3 are overlaid. The different residues in HCAR2 (green) and HCAR3 (magenta), as well as the surrounding conserved residues (white) are shown in stick representation. **g**, **h** Trajectory clustering results of acipimox (orange red) in HCAR3 (gray). Occupancy for cluster 1 (**g**) and cluster 2 (**h**) are 81.41% and 18.59%, respectively.

Of the entire hydroxycarboxylic acid receptor family, both HCAR2 and HCAR3 share relatively low homology with HCAR1, because their amino acid sequence identities are only 48.9% and 47.0%, respectively. By comparison, there is up to 96% sequence identity between HCAR2 and HCAR3, which differs by only 15 amino acid residues in most of their domains, including the N-terminus, TM1–7, ECL1–3, and ICL1–3 (Supplementary Fig. S17). Specifically, eight amino acid clusters in the TM1–7; one amino acid cluster in the ECL1; and six amino acid clusters in the ECL2 (Fig. 3a). Moreover, HCAR3 has an extended C-terminus containing 24 additional amino acids. Given that the two receptors are highly homologous, this allowed us to model the HCAR3 structure relatively accurately based on our resolved HCAR2 structures. Through analysis of conformational differences, we decided to explore the possible reasons why some agonists, such as niacin and acipimox, can bind to both HCAR2 and HCAR3, but display higher selectivity for HCAR2^13^. These findings are especially important for the development of more selective HCAR2-targeting drugs.

The homology model for HCAR3 was constructed using the acipimox-bound HCAR2 complex as a template with the SWISS-MODEL online server. Then, we performed fine mapping of the 15 residues differing in the HCAR2 and HCAR3 structures (Fig. 3b and Supplementary Fig. S18a, b). With the exception of three residues located at positions 142, 156, and 173, the remaining 12 residues were found around the coordinates of the bound ligand. In particular, 7 of 12 residues, located at positions 83, 86, 91, 103, 107, 176, and 178, were directly related to the formation of the orthosteric binding pocket (Fig. 3d, e). The differences in these seven residues not only rendered the pocket volume ~100 Å^3^ larger in HCAR3 than in HCAR2, but also generated an additional region (~358 Å^3^) at the top of the pocket. For better comparison, we overlaid their orthosteric binding pockets and subdivided them into three parts, which were defined as OBP1, OBP2, and OBP3 (Fig. 3f). As the main cavity that accommodated the ligand, the OBP1 regions of HCAR2 and HCAR3 were well overlapped, and their volumes were ~316 Å^3^. The OBP2 regions did not differ significantly, and had volumes of ~46 Å^3^ (HCAR2) and ~63 Å^3^ (HCAR3) (Supplementary Fig. S18d). This was primarily because the residues comprising OBP2, including L30^1.35^, L34^1.39^, Y87^2.64^, F277^7.36^, L280^7.39^, and Y284^7.43^, were all highly conserved. In contrast, the most remarkable difference was the extra cavity (OBP3 region) with ~84 Å^3^ in HCAR3, which was absent in HCAR2 (Supplementary Fig. S18c). There were seven residues associated with the OBP3 formation, but four differed among them: L83^2.60^, N86^2.63^, M103^3.28^, and L107^3.32^ were in HCAR2, while V83^2.60^, Y86^2.63^, V103^3.28^, and F107^3.32^ were in HCAR3. Another difference of note was the additional region of HCAR3, the formation of which was related to the replacement of the residues at positions 86, 91, 176, and 178 (Supplementary Fig. S18e). Particularly, the bulky residue W91^ECL1^ in the HCAR2 was positioned on the top of the orthosteric pocket like a lid, whereas it was substituted with a smaller residue, S91^ECL1^, in HCAR3, thus producing a large additional region composed of the N-terminus, ECL1, ECL2, TM1, TM2, and TM7. We then mutated these distinct residues in HCAR2 to the allelic residues in HCAR3 and found that six of the mutations, located at positions 86, 91, 103, 107, 176, and 178, markedly reduced the acipimox activity in HCAR2 (Fig. 3c). Consistently, the reverse mutations of these six residues in HCAR3 displayed increased agonistic activity for acipimox compared to the wild-type HCAR3. In view of these results, we speculated that the differences in the pocket volume and shape of the two receptors, especially contributed by residues at positions 86, 91, 103, 107, 176, and 178, had a significant influence on the agonist selectivity between HCAR2 and HCAR3.

To further investigate the binding differences of agonists in HCAR2 and HCAR3, we first docked acipimox into the orthosteric pocket of HCAR3. Then the MD simulations were performed to explore the time evolution of interactions between the ligands and specific residues, including niacin and acipimox in HCAR2, as well as acipimox in HCAR3 (Supplementary Fig. S12a–c). During the simulation, the hydrogen bond between S179^45.52^ and niacin (or acipimox) in HCAR2 demonstrated an impressive capability to persist in a stable state (Supplementary Fig. S12d, e). Whereas the lifetime of the hydrogen bond formed between Y284^7.43^ and the ligand in the niacin-bound HCAR2 was found to be longer compared to that in the acipimox-bound form. In comparison, acipimox failed to form a stable hydrogen bond with both S179^45.52^ and Y284^7.43^ in the HCAR3 receptor (Supplementary Fig. S12f). Analyzing the salt bridge interactions in HCAR2, we observed that niacin established a stable salt bridge interaction with R111^3.36^ throughout the 200 ns simulation, which was more stable than the salt bridge mediated by acipimox (Supplementary Fig. S12g, h). Conversely, for the acipimox in the HCAR3 binding pocket, a stable salt bridge was only observed during the last 40 ns, indicating an unstable binding mode (Supplementary Fig. S12i). We then employed the density peak method^37^ to cluster the trajectory of acipimox in HCAR3 and observed two main binding poses, in which the carboxy group direction was either toward the intracellular side (account for 18.59%) or toward the extracellular side (account for 81.41%) (Fig. 3g, h). Furthermore, the MMPBSA calculations revealed that the binding free energy of niacin in HCAR2 (−13.60 kcal/mol) surpassed that of acipimox in HCAR2 (−7.93 kcal/mol) and significantly outperformed acipimox in HCAR3 (−1.78 kcal/mol), which were consistent with the binding ability of niacin and acipimox in HCAR2 and HCAR3.

### Activation of the HCAR2 receptor

**Fig. 4:**
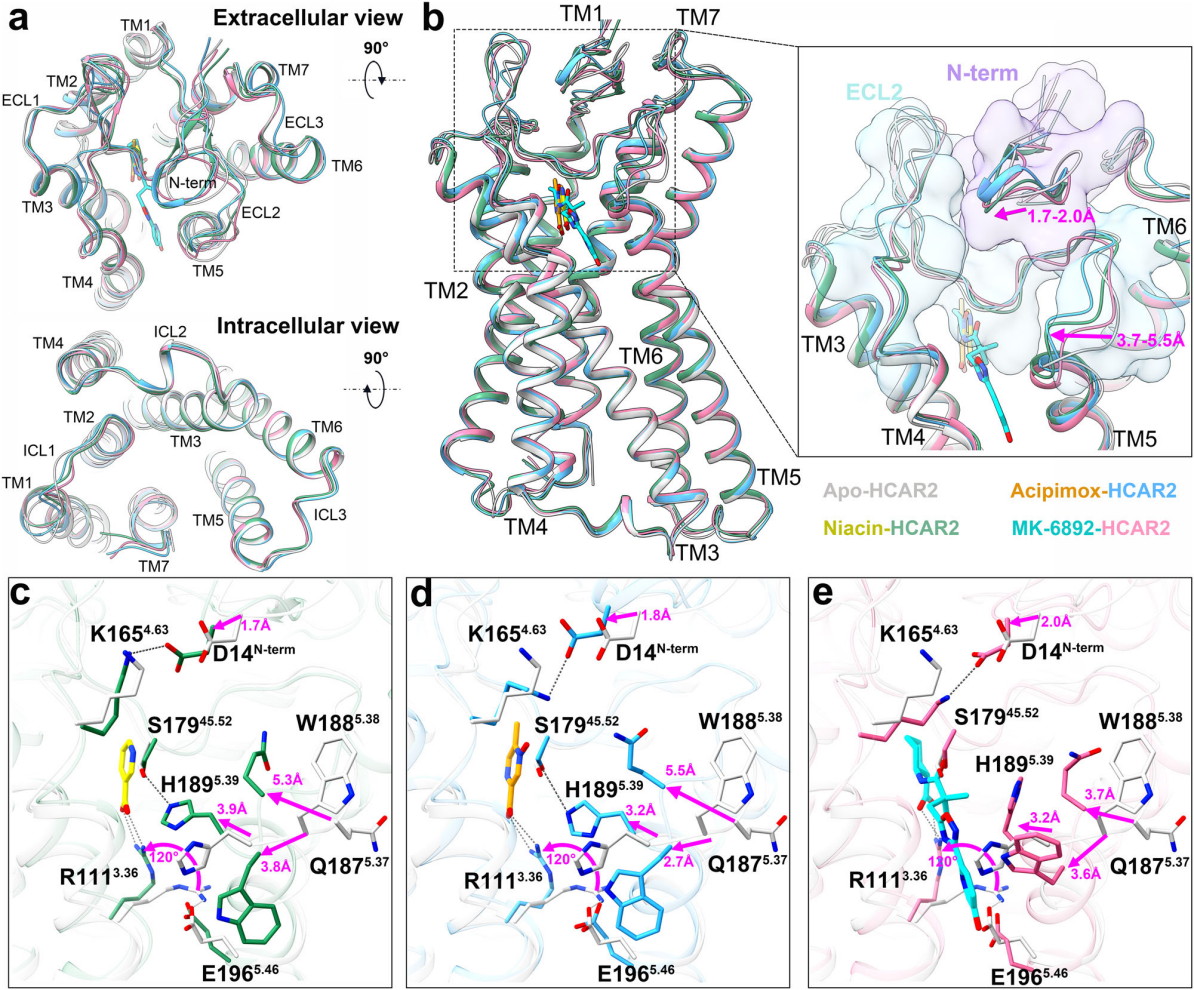
Structural comparison of HCAR2 in four active states. **a** Extracellular and intracellular views of the comparison of the apo and agonist-bound HCAR2 structures. **b** Structural differences of the N-terminus and ECL2 in the four active state HCAR2 receptors. **c**–**e** Pairwise comparisons of the apo state versus niacin- (**c**), acipimox- (**d**), and MK-6892-bound (**e**) forms. The structures of HCAR2 and agonists are colored differently. Light gray, apo-HCAR2; forest green, niacin–HCAR2; deep sky blue, acipimox–HCAR2; hot pink, MK-6892–HCAR2; blue purple, N-terminal loop; sky blue, ECL2; yellow, niacin; dark orange, acipimox; cyan, MK-6892; magenta arrow, shift with respect to the apo state; dark gray dashed lines, polar interactions.

The four active state HCAR2–Gi1 complexes enabled us to explore the activation mechanism of HCAR2. As can be clearly seen from Fig. 4a, the overall structure of the TM1–7 regions and several canonical activation-related motifs, such as P^5.50^–I^3.40^–F^6.44^, N/D^7.49^P^7.50^xxY^7.53^, and E/D^3.49^R^3.50^Y^3.51^ (where x is any residue), are highly superimposed in the apo and agonist-bound HCAR2 structures (Supplementary Fig. S19a–c). Typically, the activation motion of most class A GPCRs is triggered by a conserved residue W^6.48^, which serves as the “toggle switch” in TM6^38^. Afterward, the movement of W^6.48^ gives rise to the rearrangement of the conserved P^5.50^–I^3.40^–F^6.44^ motif and leads to the outward movement of TM6^39^. When the activation signal is propagated through the conserved N/D^7.49^P^7.50^xxY^7.53^ motif to the bottom E/D^3.49^R^3.50^Y^3.51^ motif, TM6 moves further outward to accommodate the binding of G proteins^40^. Like most class A GPCRs, these important motifs mentioned above were conserved in HCAR2, as the corresponding positions were P200^5.50^–I115^3.40^–F240^6.44^, D290^7.49^P291^7.50^xxY294^7.53^, and D124^3.49^R125^3.50^Y126^3.51^ (Supplementary Fig. S19a–c). Notably, in HCAR2, the conserved W^6.48^ was replaced by F244^6.48^, which established extensive aromatic and hydrophobic interactions with surrounding residues, including the P200^5.50^–I115^3.40^–F240^6.44^ motif and F197^5.47^ (Supplementary Fig. S19a). To our knowledge, this change is common and present in many δ-branch class A GPCRs (e.g., protease-activated receptors (PARs) and cysteinyl leukotriene receptors (CysLTRs)), because many of them have F^6.48^ instead of W^6.48^ at this position^41,42^.

Interestingly, the ligand binding seemed to make the HCAR2 structure tighter and more stable, which was predominately reflected in the N-terminus and ECL2. We performed pairwise comparisons for the apo state versus niacin-, acipimox-, and MK-6892-bound forms. Compared with the apo state, the N-terminus and ECL2 in the agonist-bound HCAR2 structures were observed to move 1.7–2.0 Å and 3.7–5.5 Å, respectively (Fig. 4b). Specifically, the movement of the N-terminus was mainly mediated by D14^N-term^, which closed the distance between the N-terminus and ECL2 by forming a salt bridge with K165^4.63^ (Fig. 4c–e). Additionally, in the apo state, the residue R111^3.36^ formed an “ionic lock” with E196^5.46^. However, the addition of an agonist disrupted this interaction and induced R111^3.36^ to rotate upward ~120° to establish a salt bridge with an acidic group of the agonist. Meanwhile, a large structural rearrangement occurred in Q187^5.37^, W188^5.38^, and H189^5.39^ of ECL2, which moved 3.7–5.5 Å, 2.7–3.8 Å, and 3.2–3.9 Å, respectively, and rotated at different degrees. Together these changes eventually led to the formation of multiple polar interactions between the agonist and R111^3.36^, Y284^7.43^, and S179^45.52^, as we described previously in niacin-, acipimox-, and MK-6892-bound complexes (Fig. 2c–e). Thus, the ligand binding, to some extent, increased the structural stability of HCAR2 by tethering TM3, TM7, and ECL2 together. It was worth noting that the conformational change of residue H189^5.39^ had been regarded as important for the formation of the orthosteric binding pocket in the MK-6892-bound form (Fig. 2f). Here, we further observed that in the niacin- and acipimox-bound forms, H189^5.39^ extended to the ligand binding orientation and formed a hydrogen bond with S179^45.52^, making the ECL2 conformation more stable (Fig. 4c, d).

Further study is needed to understand how the ECL2 acts as a built-in “agonist” to activate the HCAR2 receptor. We compared the apo state of HCAR2 with the recently published inactive state (PDB: 7ZLY)^25^ (Supplementary Fig. S20a). Surprisingly, a hydrophobic residue F180^ECL2^ in ECL2 was observed to rotate downward ~170° (Supplementary Fig. S20b), thus deeply inserting into the orthosteric pocket and packing tightly into a local aromatic environment formed by the residues F276^7.35^, F277^7.36^, and F193^5.43^ (Supplementary Fig. S21a, b). In contrast, the key residue R111^3.36^ in the apo state did not change significantly relative to the inactive state. In addition, several substantial conformational changes that related to HCAR2 activation were also noted in the apo state, which were mainly reflected in the shifts of TM5, TM6, and key motifs, such as P^5.50^–I^3.40^–F^6.44^, N/D^7.49^P^7.50^xxY^7.53^, and E/D^3.49^R^3.50^Y^3.51^ (Supplementary Fig. S20c–e). Afterwards, the single-point mutations of F180^ECL2^, F276^7.35^, F277^7.36^, and F193^5.43^ were conducted, which reduced the signaling activity of HCAR2 to varying degrees (Supplementary Fig. S21c). Given this, we considered that the residues F180^ECL2^, F276^7.35^, F277^7.36^, and F193^5.43^ likely played crucial roles in the self-activation of HCAR2.

### Interfaces between the HCAR2 receptor and Gi1

**Fig. 5:**
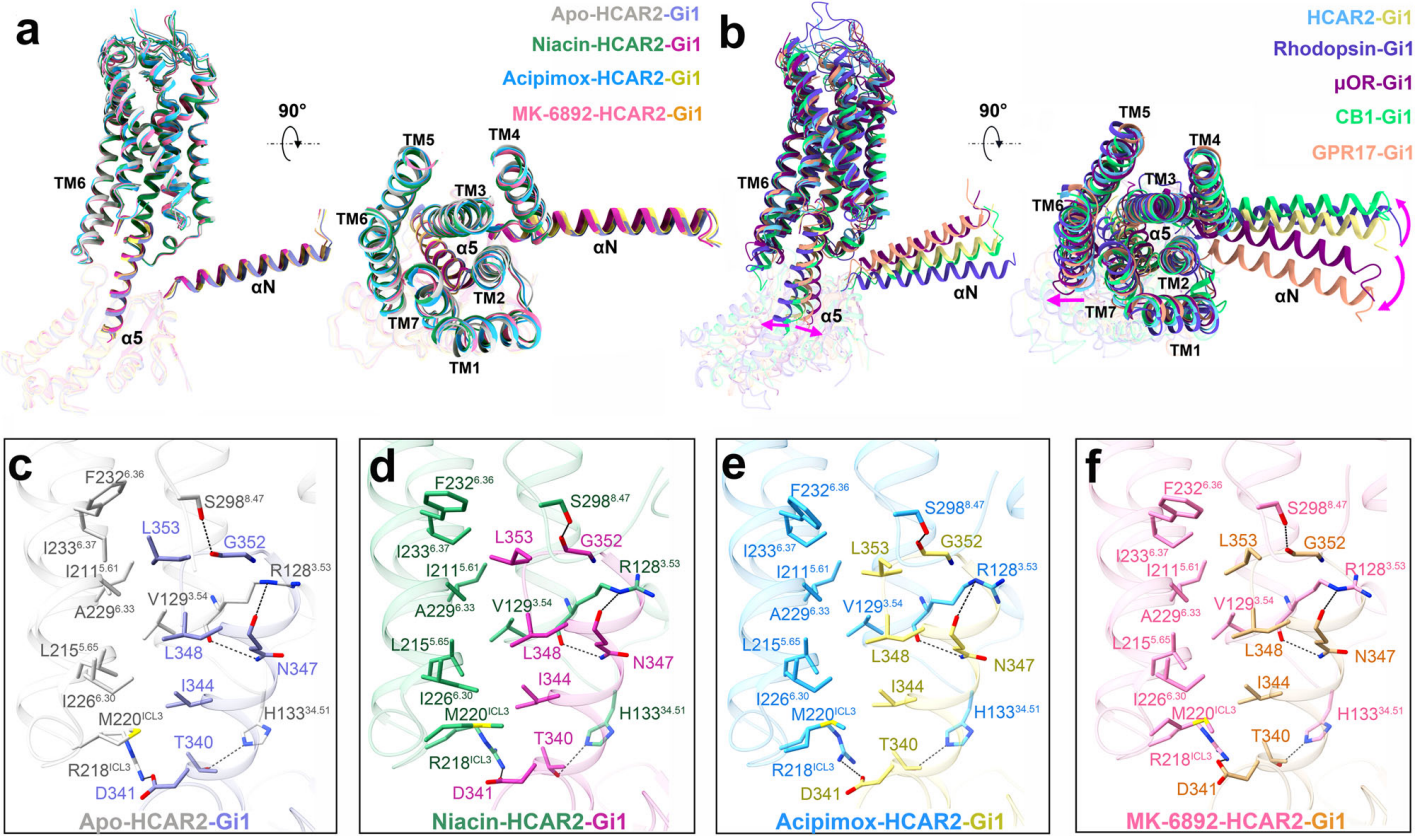
Analysis of the HCAR2–Gi1 interface and comparison with class A GPCRs. **a** Comparison of the HCAR2–Gi1 complex with or without an agonist. **b** Superimposition of the receptor G protein coupling interfaces for HCAR2–Gi1, rhodopsin–Gi1 (PDB: 6CMO, slate blue), μOR–Gi1 (PDB: 6DDE, dark magenta), CB1–Gi1 (PDB: 6N4B, turquoise), and GPR17–Gi1 (PDB: 7Y89, dark salmon). **c**–**f** Interactions of HCAR2 with the α5 helix of Gαi. The structures of HCAR2 and Gi1 are colored differently. Light gray and purple, apo-HCAR2–Gi1; forest green and plum, niacin–HCAR2–Gi1; deep sky blue and light yellow, acipimox–HCAR2–Gi1; hot pink and orange, MK-6892–HCAR2–Gi1; dark gray dashed lines, polar interactions; magenta arrow, shift with respect to HCAR2–Gi1.

The complex structures of HCAR2–Gi1 with or without agonists showed almost the same G protein coupling interface. As depicted in Fig. 5a, the interactions between HCAR2 and Gi1 were mainly mediated by the α5 helix of the Gαi subunit and the receptor cores comprised of TM1–3, TM5, TM6, ICL2, and ICL3. The αN helix of the Gαi subunit here had almost no direct interaction with the receptor. Then, the HCAR2–Gi1 complex was aligned with several canonical class A GPCR complexes, such as rhodopsin^43^, μ-opioid receptor (μOR)^44^, and cannabinoid receptor 1 (CB1)^45^, as well as self-activated GPR17^32^ (Fig. 5b). We observed that the major differences occurred in the relative positions and orientations of the α5 and αN helices, as well as the shift of TM6. For example, HCAR2 had a much less pronounced outward movement at the cytoplasmic end of TM6 compared to the representative GPCRs. Relative to the HCAR2–Gi1 complex, the α5 and αN helices rotated clockwise ~3.5 and ~7.6 Å in rhodopsin–Gi1; anticlockwise ~4.9 and ~8.1 Å in μOR–Gi1; clockwise ~1.4 and ~3.3 Å in CB1–Gi1; and anticlockwise ~2.7 and ~13.1 Å in GPR17–Gi1, respectively (Supplementary Fig. S22a–d). Subsequently, we overlaid these receptors and found that their ICL2 and ICL3 regions differed greatly, which were involved in the binding to the G protein (Supplementary Fig. S22e). Furthermore, the G protein binding pocket analysis also revealed that the pocket’s size, shape, hydrophilic, and hydrophobic properties showed clear differences (Supplementary Fig. S22f). Thus, we considered that the potential basis for the different shifts of the α5 and αN helices was mostly attributed to the structural differences of these receptors.

Similar to many other Gi-bound class A GPCRs, the C-terminus of the α5 helix was amphipathic and inserted into the cytoplasmic cavity of HCAR2 by forming extensive hydrophobic and electrostatic interactions, as well as hydrogen bonds (Fig. 5c–f and Supplementary Fig. S23). Three large hydrophobic side chains L353, L348, and I344 of the α5 helix embedded in a hydrophobic groove of HCAR2 formed by TM3, TM5/6, and ICL3 residues (TM3: V129^3.54^; TM5: I211^5.61^ and L215^5.65^; TM6: I226^6.30^, A229^6.33^, F232^6.36^, and I233^6.37^; and ICL3: M220^ICL3^), forming extensive hydrophobic interactions. Electrostatic interactions were also crucial for the stabilization of the binding mode between HCAR2 and Gi1. HCAR2 displayed a highly positive charge at the cytoplasmic end of ICL3, contributed by R218^ICL3^. Correspondingly, the α5 helix C-terminus was highly negatively charged, contributed by D341, and ultimately established a salt bridge between α5 and HCAR2. In addition, the residues R128^3.53^, H133^34.51^, and S298^8.47^ of HCAR2 formed well-defined hydrogen bonds with N347, T340, and G352 of the α5 helix, respectively (Fig. 5c–f). Together, all these findings clarified the Gi1 coupling features of HCAR2 and provided a greater understanding of the G protein coupling mechanism.

## Discussion

In recent decades, a series of HCAR2 agonists have been successfully discovered and four of them, including niacin, acipimox, acifran, and MMF, have been approved for clinical treatment of cardiovascular and neurological disorders, such as dyslipidemia, atherosclerosis, and relapsing multiple sclerosis^13^. Despite the favorable clinical efficacy, all four drugs can cause the unwanted side effect of cutaneous flushing^46,47^. It is known that flushing is a cutaneous vasodilation accompanied by a burning sensation mainly affecting the upper body and face^4^. There is good evidence that cutaneous flushing is associated with the activation of HCAR2 at Langerhans cells and keratinocytes, as well as the subsequent release of vasodilatory prostaglandins^46^. In light of this, some highly subtype-specific HCAR2 agonists with fewer side effects have been developed, especially MK-6892. In vivo rat and dog experiments demonstrated that MK-6892 displayed excellent therapeutic index compared to niacin in free fatty acid reduction, but significantly relieved the flushing effect^22^. Unfortunately, it is still unknown what the similarities and differences in the recognition mechanisms and binding modes between the approved drugs and subtype-specific agonist MK-6892 are. Given this, we reported four cryo-EM structures of HCAR2–Gi1 complexes in the apo and niacin-, acipimox-, MK-6892-bound forms.

Previous studies had reported several GPCRs with self-activation by adopting ECL2 as a built-in “agonist”, such as GPR52, GPR17, and BILF1^30–32^. For the apo-HCAR2 structure, we showed that HCAR2 could also form a stable complex with Gi1 protein in the absence of an agonist. The unique extracellular architecture of the ECL2 motif had a key role in the self-activation of HCAR2. Particularly, a hydrophobic residue F180^ECL2^ in ECL2 was thought to be most important, which was observed to rotate considerably and deeply insert into the orthosteric pocket relative to the inactive state. In fact, for all three hydroxycarboxylic acid receptors (HCAR1–3), the key residue mentioned above in ECL2 was conserved (HCAR1: F168^ECL2^; HCAR2: F180^ECL2^; HCAR3: F180^ECL2^), and we had purified and obtained their stable complexes without requiring ligand binding (data not shown). Thus, further research is necessary to understand the physiological significance of the self-activation of the HCAR family.

Combining the niacin- and acipimox-bound HCAR2 structures, as well as the molecular simulation results, we found that many agonists, including butyrate, β-OHB, niacin, acipimox, acifran, and MMF, all adopted similar binding poses and bound to HCAR2 by directly interacting with three key residues, R111^3.36^, S179^45.52^, and Y284^7.43^, in the orthosteric binding pocket. Among them, the salt bridge between an acidic group and basic R111^3.36^ was considered as the most important interaction. Based on this, the detailed recognition mechanisms of HCAR2 for endogenous ligands, approved drugs, and subtype-specific agonist were revealed, which were critical for understanding how these agonists exerted their anti-lipolytic and anti-inflammatory functions. More importantly, the general pharmacophore features that may fit most of the agonists recognized by HCAR2 were summarized.

Compared with niacin and acipimox, the highly subtype-specific MK-6892 had several unique groups, resulting in a larger and more complex chemical structure. To accommodate the bulky MK-6892, substantial conformation changes of W188^5.38^, H189^5.39^, and M192^5.42^ led to the formation of an extended binding pocket in the MK-6892–HCAR2 complex. As a result, MK-6892 not only formed similar interactions with R111^3.36^, Y284^7.43^, and S179^45.52^ as niacin and acipimox in the orthosteric pocket, but also introduced an additional polar interaction with Q112^3.37^ in the extended binding pocket. On one hand, this well explained the reason for the higher affinity of HCAR2 for MK-6892 than for niacin and acipimox^22^. On the other hand, we speculated that the differences in the binding modes of MK-6892 and of niacin and acipimox might differentially activate downstream signaling pathways, thus selectively eliciting the therapeutic, anti-lipolytic pathway, while avoiding the activation of the flush-inducing pathway. To further explore this, we measured the coupling preference of HCAR2 to several G protein subtypes (including Gi, Gs, and Gq) as well as β-arrestin recruitment, when bound to different agonists (Supplementary Fig. S24). In response to the activation mediated by niacin, acipimox, and MK-6892, both the β-arrestin and Gi-coupled signaling could be observed, while the coupling to Gs or Gq was negligible. Notably, compared to niacin and acipimox, MK-6892 exhibited higher efficacy either in Gi coupling or β-arrestin recruitment, which was similar to that reported in a recent study^25^. However, some previous studies suggested that the weak β-arrestin activation of HCAR2 agonists (such as MK-0354) was usually preferred to reduce the cutaneous flushing^48^. Therefore, we need more efforts to study the correlation between the β-arrestin signaling and skin flushing in the future.

As a close relative of HCAR2, HCAR3 is considered to be the result of a recent gene duplication present in humans and higher primates, such as chimpanzees^1,49^. Unlike HCAR2, the natural ligand of HCAR3 is 3-hydroxyoctanoic acid, which exerts anti-lipolytic activity in the human body^49^. Interestingly, HCAR2 is not activated by 3-hydroxyoctanoic acid, and the HCAR2 natural ligands β-OHB and butyrate have no activity toward HCAR3 as well^50^. Furthermore, some agonists, such as niacin and acipimox, can bind to both receptors, but display higher selectivity for HCAR2^13^. To explore the ligand selectivity differences between HCAR2 and HCAR3, we constructed a model of HCAR3 relatively accurately using the acipimox-bound HCAR2 complex as a template. Structural alignment suggested that although only 15 differential residues were observed in the major domains of HCAR2 and HCAR3, six of them, especially at positions 86, 91, 103, 107, 176, and 178, significantly affected the volume and shape of the orthosteric binding pockets: (1) the pocket volume of HCAR3 was larger than that of HCAR2; (2) an additional region was generated on the top of the HCAR3 pocket. After clustering the MD trajectory of acipimox in HCAR3, two main binding poses were observed, in which the carboxy group of acipimox was either toward the intracellular side or toward the extracellular side. Meanwhile, the salt bridge and hydrogen bond networks of acipimox in HCAR3 were less stable than those in HCAR2, which were consistent with the calculated values of binding free energy. We conjectured that the smaller pocket volume of HCAR2 might be more favorable for precise positioning and binding of acipimox to the surrounding residues, thus forming stable interactions. Our results were also confirmed by the study of Ahmed et al., in which the residues at positions 86, 103, and 107 were considered to be critically involved in forming the selective binding site in HCAR3^50^. To get more details on the precise interactions between ligands and HCAR3, the studies of cryo-EM structures of agonist-bound HCAR3 are in progress. Overall, our structural analysis provides a deep understanding of the ligand recognition, selectivity, activation, and G protein coupling mechanism of HCAR2, which is important for the design of HCAR2-targeting drugs with greater efficacy, higher selectivity, and fewer or no side effects.

## Materials and methods

### Expression and purification of the HCAR2–Gi1 complex

Wild-type human HCAR2 was cloned into pFastBac vector (Gibco) with N-terminal hemagglutinin (HA) signal sequence, Flag tag, and HRV 3C protease site, as well as a C-terminal His tag. Dominant-negative Gαi1 (DNGαi1) with mutations (G203A and A326S) was constructed in the same manner as HCAR2. The pFastBac Dual vector (Gibco) was used to construct the Gβ_1_γ_2_ expression vector. The co-expression of HCAR2, DNGαi1, and Gβ_1_γ_2_ proteins was achieved using the Bac-to-Bac baculovirus expression system in *Spodoptera frugiperda* Sf9 cells (Invitrogen). Cells were grown in suspension at 27 °C to a density of 4 × 10^6^ cells ml^−1^ and infected with virus at a ratio of 10:10:1 (HCAR2: DNGαi1: Gβ_1_γ_2_). After 48 h of infection, the cells were collected by centrifugation and then stored at −80 °C until use.

To obtain the HCAR2–Gi1 complex, cell pellets were thawed and suspended in lysis buffer (10 mM HEPES, pH 7.5, 0.5 mM EDTA) and supplemented with 50 μM niacin (MCE HY-B0143), acipimox (MCE HY-B0283), MK-6892 (MCE HY-10680), or without an agonist. Then, the sample was rotated at 4 °C for 60 min to induce HCAR2–Gi1 complex formation. A dounce homogenizer was used to homogenize and collect cell membranes in a solubilization buffer (20 mM, HEPES, pH 7.5, 100 mM NaCl, 50 µM agonist, 10% glycerol, 1% (w/v) n-Dodecyl-b-D-maltoside (DDM), 0.1% (w/v) cholesteryl hemisuccinate (CHS), 0.2 µg/mL leupeptin, 100 µg/mL benzamidine, 10 mM MgCl_2_, 5 mM CaCl_2_, 1 mM MnCl_2_, 100 μU/mL lambda phosphatase (NEB), and 25 μU/mL apyrase (NEB)). After incubating at 4 °C for 2 h, the supernatant was collected by centrifugation and then incubated with anti-Flag M1 antibody affinity resin at 4 °C for 1 h. The M1 resin was washed with wash buffer (20 mM, HEPES, pH 7.5, 100 mM NaCl, 50 µM agonist, 0.1% DDM, 0.01% CHS, and 2 mM CaCl_2_). The buffer was changed from DDM to lauryl maltose neopentyl glycol (LMNG) using a stepwise process. Afterward, the M1 resin was washed with the LMNG buffer (20 mM HEPES, pH 7.5, 100 mM NaCl, 50 µM agonist, 0.01% (w/v) LMNG, 0.001% CHS, and 2 mM CaCl_2_). The HCAR2–Gi1 complex was eluted with elution buffer (20 mM HEPES, pH 7.5, 100 mM NaCl, 50 µM agonist, 0.00075% LMNG, 0.00025% (w/v) glycol-diosgenin (GDN), 0.0001% CHS, 5 mM EDTA, and 200 µg/mL synthesized Flag peptide). The eluted protein was concentrated and incubated with the antibody fragment scFv16 for 2 h on ice at a molar ratio of 1:1.5^51^. A Superdex 200 Increase 10/300 column (GE Healthcare) was pre-equilibrated with buffer (20 mM, HEPES, pH 7.5, 100 mM NaCl, 0.00075% LMNG, 0.00025% GDN, 0.0001% CHS, and 50 μM agonist) and then used to further purify the complex. The obtained pure HCAR2–Gi1–scFv16 complex was concentrated in an ultrafiltration tube and flash-frozen in liquid nitrogen until further use.

### GTPase-Glo assay

To perform the GTPase-Glo assay, the HCAR2 protein was purified as described above. Then we initiated the GTPase reaction by mixing Gi1 and HCAR2 in 5 µL of reaction buffer (20 mM HEPES, pH 7.5, 100 mM NaCl, 0.02% LMNG, 1 mM MgCl_2_, 5 µM GTP, 5 µM GDP, with or without 50 μM test agonist) in a 384-well plate. The final concentrations of HCAR2 and Gi1 were 4 µM and 0.5 µM, respectively. Gi1 alone was set as a reference in every independent experiment. At room temperature (22–25 °C), the GTPase reaction was incubated for 2 h. Then 5 µL of reconstituted 1× GTPase-Glo reagent (Promega) was added, mixed briefly, and incubated with shaking for 30 min to convert the remaining GTP into ATP. Afterward, to convert the ATP into luminescent signals, we added 10 µl of detection reagent (Promega) to the system, which was incubated in the 384-well plate for 5–10 min at room temperature. The Multimode Plate Reader (PerkinElmer EnVision 2105) luminescence counter was used to quantify the luminescence intensity. Data were analyzed using GraphPad Prism 9.0.

### Cryo-grid preparation and EM data collection

To prepare the cryo-EM sample, the 100 Holey Carbon film (Au, 300 mesh, N1-C14nAu30-01) was pre-discharged with Tergeo-EM plasma cleaner. Then, 3 μL of the purified HCAR2–Gi1–scFv16 complex was applied to the grid. At 10 °C and 100% humidity, the sample was incubated for 3 s and blotted for 2 s using the freezing plunger Vitrobot I (Thermo Fisher Scientific, USA). Grids were quickly frozen in liquid ethane cooled by liquid nitrogen and stored in liquid nitrogen until checked. We used the 300 kV Titan Krios Gi3 microscope (Thermo Fisher Scientific FEI, the Kobilka Cryo-EM Center of the Chinese University of Hong Kong, Shenzhen) to check the grids and collect the cryo-EM data of the HCAR2–Gi1–scFv16 complex. The Gatan K3 BioQuantum camera at a magnification of 105,000 was used to record movies, and the pixel size was 0.83–0.85 Å. We used the GIF-quantum energy filter (Gatan, USA) to exclude the inelastically scattered electrons. The slit width of the filter was set to 20 eV. The movie stacks were automatically acquired with the defocus range from −1.1 to −2.0 μm. The exposure time was 2.5 s, with frames collected for a total of 50 frames (0.05 s/frame) per sample. The dose rate was 21.2 e/pixel/s. SerialEM 3.7 was used for semiautomatic data acquisition.

### Image processing and 3D reconstructions

The general strategy in image processing follows the method in a hierarchical way as described^52^. Data binned by 4 times is used for micrograph screening and particle picking. The data with 2-time binning is used for particle screening and classification. The particle after initial cleaning was subjected to extraction from the original clean micrograph and the resultant dataset was used for final cleaning and reconstruction. Raw movie frames were aligned with MotionCor2^53^ using a 9 × 7 patch and the contrast transfer function (CTF) parameters were estimated using Gctf and ctf in JSPR^54^. Only the micrographs with consistent CTF values including defocus and astigmatism were kept for following image processing. For HCAR2–Gi1–scFv16 protein, 3456 movies were processed by cryoSPARC v4.1.1^55^. Each movie stack was aligned with patch motion correction. A total of 2,947,103 particles were extracted with auto-picking. After three rounds of 2D classification, the number of good particles was reduced to 515,376. The number of particles was further reduced to 311,666 by 3D classification and Ab-initio reconstruction. A 3.28 Å resolution density map at FSC 0.143 was obtained when the initial map of the particles was processed with homogeneous refinement, non-uniform refinement, and local refinement. For HCAR2–Gi1–scFv16 protein with niacin, 2961 movies were processed by cryoSPARC v4.1.1. Each movie stack was aligned with patch motion correction. A total of 3,191,801 particles were extracted with the auto-picking. After three rounds of 2D classification, the number of good particles was reduced to 1,563,889. The number of particles was further reduced to 879,036 by 3D classification and Ab-initio reconstruction. A 2.69 Å resolution density map at FSC 0.143 was obtained when the initial map of the particles was processed with homogeneous refinement, non-uniform refinement, and local refinement. For HCAR2–Gi1–scFv16 protein with acipimox, 1706 movies were processed by cryoSPARC v4.1.1. Each movie stack was aligned with patch motion correction. A total number of 1,328,380 particles were extracted with the auto-picking. After three rounds of 2D classification, the number of good particles was reduced to 454,521. The number of particles was further reduced to 221,940 by 3D classification and Ab-initio reconstruction. A 3.23 Å resolution density map at FSC 0.143 was obtained when the initial map of the particles was processed with homogeneous refinement, non-uniform refinement, and local refinement. For HCAR2–Gi1–scFv16 protein with MK-6892, 2518 movies were processed by cryoSPARC v4.1.1. Each movie stack was aligned with patch motion correction. A total number of 1,988,362 particles were extracted with the auto-picking. After three rounds of 2D classification, the number of good particles was reduced to 558,862. The number of particles was further reduced to 291,441 by 3D classification and Ab-initio reconstruction. A 3.25 Å resolution density map at FSC 0.143 was obtained when the initial map of the particles was processed with homogeneous refinement, non-uniform refinement, and local refinement.

### Model building and refinement

Alphafold (https://alphafold.ebi.ac.uk/) was used to predict the human HCAR2 structure, which was used as a template to build the HCAR2–Gi1–scFv16 complex model. Gi–scFv16 was built using the Gi1 heterotrimer from the FPR2–Gi cryo-EM structure (PDB: 6OMM) as the template^56^. All models were subsequently docked into the density maps using UCSF Chimera, followed by iterative manual adjustment and rebuilding in COOT 0.9.7 and phenix.realspace refinement. The final refinement model statistics were validated by Phenix. The molecular docking of agonists with HCAR2 and HCAR3 was performed using the triangle matching method implemented in the MOE2019.01 software. We performed 3 independent docking runs and 50 conformers were obtained in each case. The possible conformations were ranked based on their London dG scores. The GBVI/WSA dG scoring function was used to further refine the results, and the conformation with the maximum score was selected. The molecular graphics figures were presented using UCSF Chimera, UCSF ChimeraX, and PyMOL. The final refinement statistics were validated using Molprobity and shown in Supplementary Table S1. Besides, the residues with sidechain deletion due to the lack of density were shown in Supplementary Table S2.

### MD simulation

To complete the missing parts of Gαi in the solved structure. We used the structure of heterothermic Gi protein bound with GDP (PDB: 7RKY) as a template to perform homology modeling using the MODELLER program^57^. The Membrane Builder module in CHARMM-GUI server^58^ was used to prepare the simulation inputs, including a membrane of pre-equilibrated (310 K) POPC lipids based on the OPM database alignment^59^, TIP3P solvent with 0.15 M Na^+^/Cl^–^ ions, and the CHARMM36m forcefield^60^. The force field of the ligands was generated by the CGenFF program^61^. All MD simulations were performed using GROMACS-2019.4^62^. The CHARMM36m forcefield was used to describe the interactions in the system. Energy minimization was performed for 5000 steps by the steepest descent algorithm. Then a 250 ps NVT simulation was performed at 310 K for solvent equilibration, followed by a 1 ns NPT equilibration to 1 atm using the Berendsen barostat^63^. All MD simulations were performed with a time-step of 1 fs. Long-range electrostatic interactions were treated by the particle-mesh Ewald method^64^. The short-range electrostatic and van der Waals interactions both used a cutoff of 10 Å. All bonds were constrained by the LINCS algorithm^65,66^. Here, MD simulations were started from the solved structures of the acipimox-bound HCAR2 and the niacin-bound HCAR2, as well as the modeled structure of the acipimox-bound HCAR3. Simulation runs for 200 ns. The trajectory was analyzed by the python package MDtraj^66^ and the last 1 ns trajectory was employed to calculate the binding free energy using the gmx_MMPBSA method^67^.

### cAMP assay

The Gi/o-cAMP and Gs assays were conducted using a cAMP-Gi/o kit (Cisbio, 62AM9PEB) and cAMP-Gs kit (Cisbio, 62AM4PEB), respectively. The Gi/o-cAMP assay was performed as follows. Wild-type HCAR2 and its mutants were cloned into a pcDNA3.1 vector. Before transfection, HEK293 cells (ATCC CRL-1573) were seeded in 24-well culture plates at a density of 70%–90% cells per well. Then the cells were transiently transfected with the plasmid using Lipofectamine 3000 reagents (Invitrogen, L3000). After 36 h, the culture media was removed, and the cells were washed with PBS buffer. The transfected cells were then plated into 384-well plates (4000/well) in a stimulation buffer and treated with 20 μΜ forskolin, 500 μM IBMX, and the test agonist for 30 min at 37 °C. Thereafter, 5 µl cAMP Eu-cryptate reagent and 5 μl of anti-cAMP-d2 working solution were added to the 384-well plates and incubated for 1 h^68^. Fluorescence signals were detected at 620/665 nm using the Multimode Plate Reader (PerkinElmer EnVision 2105)^69^. Data were analyzed with GraphPad Prism 9.0. The experiments were performed in triplicate. The experimental method of the Gs-cAMP assay was similar to that of the Gi/o-cAMP assay without addition of forskolin.

### Inositol phosphate (IP) accumulation assay

The IP1 accumulation was conducted using an IP-One Gq assay kit (Cisbio Bioassays, 62IPAPEC). In brief, the harvested HEK293 cells transfected with pcDNA3.1-HCAR2 were plated into 384-well plates (7000/well) and treated with different concentrations of test agonist at 37 °C for 70 min. Then 3 μL cryptate-labeled anti-IP1 monoclonal antibody and 3 μL d2-labeled IP1, which were pre-diluted in Lysis Buffer (1:20), were added to the wells, and incubated at room temperature for 1 h. The plates were then read by the Multimode Plate Reader (PerkinElmer EnVision 2105) at 620/665 nm. The experiments were performed in triplicate.

### NanoBiT-based β-arrestin recruitment assay

For NanoBiT-based assay, HEK293 cells were seeded in a 24-well plate, incubated for 16 h and then co-transfected with HCAR2-SmBiT (400 ng/well) and LgBiT-β-arrestin1 (200 ng/well) by Lipofectamine 3000 for 24 h at 37 °C. Cells were harvested and seeded on white 384-well plates (20,000/well). The substrate of coelenterazine H was added to the plates at a final concentration of 10 μM. After 25 min incubation at 37 °C, different concentrations of test agonist were added. Cells were rested for 30 min at 37 °C. Then the luminescence was detected for 30 min. The plate was measured for baseline luminescence using an Envision plate reader (PerkinElmer, Boston, MA). The experiments were performed in triplicate.

### SPR measurement

The binding affinity of the wild-type HCAR2 and its mutants R111^3.36^A, Q112^3.37^A, S179^45.52^A, and Y284^7.43^A for niacin, acipimox, and MK-6892 were measured by using the Biacore X100 system in a running buffer containing 2 mM HEPES (pH 7.4), 10 mM NaCl, and 5% (v/v) DMSO. Purified wild-type HCAR2 and its mutants were immobilized on the surface of a CM5 sensor chip using the amine-coupling procedure at pH 4.5. Varying concentrations of agonist diluted in running buffer were injected as analytes for 100 s, as the period of association. Subsequently, the running buffer was alternatively perfused over the chip to allow the bound agonist to undergo a 50 s period of disassociation. Sensorgrams were recorded in real-time and analyzed in the Biacore X100 system for the calculation of binding affinity (*K*_D_).

### Cell surface expression testing

Expression levels of HCAR2 plasmid in HEK293 cells were determined by flow cytometry analysis and used to normalize the cAMP level. Specifically, the transfected cells were blocked with 5% BSA at room temperature for 15 min and labeled with anti-FLAG antibody (1:100, Thermo Fisher Scientific) at 4 °C for 1 h. After washing with PBS buffer, the cells were incubated with anti-mouse Alexa 488-conjugated secondary antibody (1:300, Beyotime) at 4 °C in the dark for 1 h. Each sample was counted with ~10,000 cellular events. The fluorescent intensity was quantified by a BD Accuri™ C6 Plus flow cytometer. The experiments were performed in triplicate. Data were analyzed using GraphPad Prism 9.0 and presented as the mean ± SEM.

### Supplementary information

Supplementary materials

PDB-apo

PDB-niacin

PDB-acipimox

PDB-MK6892

cryo-EM map-apo

cryo-EM map-niacin

cryo-EM map-acipimox

cryo-EM map-MK6892

## Data Availability

The data supporting this study are available from the corresponding author upon reasonable request. The 3D cryo-EM density maps of the niacin-, acipimox-, MK-6892-bound, and apo HCAR2–Gi–scFv16 complexes have been deposited in the Electron Microscopy Data Bank database under accession codes EMD-35483, EMD-35484, EMD-35485, and EMD-35463, respectively. The atomic coordinates for the atomic models of the niacin-, acipimox-, MK-6892-bound, and apo HCAR2–Gi–scFv16 complexes generated in this study have been deposited in the Protein Data Bank database under accession codes 8IJA, 8IJB, 8IJD, and 8IJ3, respectively.

## References

[1] Offermanns S (2011). International union of basic and clinical pharmacology. LXXXII: nomenclature and classification of hydroxy-carboxylic acid receptors (GPR81, GPR109A, and GPR109B). Pharmacol. Rev..

[2] Taggart AKP (2005). (D)-beta-Hydroxybutyrate inhibits adipocyte lipolysis via the nicotinic acid receptor PUMA-G. J. Biol. Chem..

[3] Wise A (2003). Molecular identification of high and low affinity receptors for nicotinic acid. J. Biol. Chem..

[4] Wanders D, Judd RL (2011). Future of GPR109A agonists in the treatment of dyslipidaemia. Diabetes Obes. Metab..

[5] Graff EC, Fang H, Wanders D, Judd RL (2016). Anti-inflammatory effects of the hydroxycarboxylic acid receptor 2. Metabolism.

[6] Thangaraju M (2009). GPR109A is a G-protein-coupled receptor for the bacterial fermentation product butyrate and functions as a tumor suppressor in colon. Cancer Res..

[7] Senior B, Loridan L (1968). Direct regulatory effect of ketones on lipolysis and on glucose concentrations in man. Nature.

[8] Gille A, Bodor ET, Ahmed K, Offermanns S (2008). Nicotinic acid: pharmacological effects and mechanisms of action. Annu. Rev. Pharmacol. Toxicol..

[9] Offermanns S (2017). Hydroxy-carboxylic acid receptor actions in metabolism. Trends Endocrinol. Metab..

[10] Lukasova M, Hanson J, Tunaru S, Offermanns S (2011). Nicotinic acid (niacin): new lipid-independent mechanisms of action and therapeutic potentials. Trends Pharmacol. Sci..

[11] Taing K, Chen L, Weng H-R (2023). Emerging roles of GPR109A in regulation of neuroinflammation in neurological diseases and pain. Neural Regen. Res..

[12] Karunaratne TB (2020). Niacin and butyrate: nutraceuticals targeting dysbiosis and intestinal permeability in Parkinson’s disease. Nutrients.

[13] Offermanns S (2014). Free fatty acid (FFA) and hydroxy carboxylic acid (HCA) receptors. Annu. Rev. Pharmacol. Toxicol..

[14] Altschul R, Hoffer A, Stephen JD (1955). Influence of nicotinic acid on serum cholesterol in man. Arch. Biochem. Biophys..

[15] Lukasova M (2011). Nicotinic acid inhibits progression of atherosclerosis in mice through its receptor GPR109A expressed by immune cells. J. Clin. Invest..

[16] Wu BJ (2010). Evidence that niacin inhibits acute vascular inflammation and improves endothelial dysfunction independent of changes in plasma lipids. Arterioscler. Thromb. Vasc. Biol..

[17] Moutinho M (2022). The niacin receptor HCAR2 modulates microglial response and limits disease progression in a mouse model of Alzheimer’s disease. Sci. Transl. Med..

[18] Vosper H (2009). Niacin: a re-emerging pharmaceutical for the treatment of dyslipidaemia. Br. J. Pharmacol..

[19] Tang H (2008). The psoriasis drug monomethylfumarate is a potent nicotinic acid receptor agonist. Biochem. Biophys. Res. Commun..

[20] Parodi B (2015). Fumarates modulate microglia activation through a novel HCAR2 signaling pathway and rescue synaptic dysregulation in inflamed CNS. Acta Neuropathol..

[21] Soudijn W, van Wijngaarden I, Ijzerman AP (2007). Nicotinic acid receptor subtypes and their ligands. Med. Res. Rev..

[22] Shen HC (2010). Discovery of a biaryl cyclohexene carboxylic acid (MK-6892): a potent and selective high affinity niacin receptor full agonist with reduced flushing profiles in animals as a preclinical candidate. J. Med. Chem..

[23] Sprecher D (2015). Discovery and characterization of GSK256073, a non-flushing hydroxy-carboxylic acid receptor 2 (HCA2) agonist. Eur. J. Pharmacol..

[24] Palani A (2012). Discovery of SCH 900271, a potent nicotinic acid receptor agonist for the treatment of dyslipidemia. ACS Med. Chem. Lett..

[25] Yang Y (2023). Structural insights into the human niacin receptor HCA2-Gi signalling complex. Nat. Commun..

[26] Zhao C (2023). Biased allosteric activation of ketone body receptor HCAR2 suppresses inflammation. Mol. Cell.

[27] Irukayama-Tomobe Y (2009). Aromatic D-amino acids act as chemoattractant factors for human leukocytes through a G protein-coupled receptor, GPR109B. Proc. Natl. Acad. Sci. USA.

[28] Kapolka NJ, Isom DG (2020). HCAR3: an underexplored metabolite sensor. Nat. Rev. Drug Discov..

[29] Jung J-K (2007). Analogues of acifran: agonists of the high and low affinity niacin receptors, GPR109a and GPR109b. J. Med. Chem..

[30] Lin X (2020). Structural basis of ligand recognition and self-activation of orphan GPR52. Nature.

[31] Tsutsumi N (2021). Structural basis for the constitutive activity and immunomodulatory properties of the Epstein-Barr virus-encoded G protein-coupled receptor BILF1. Immunity.

[32] Ye F (2022). Cryo-EM structure of G-protein-coupled receptor GPR17 in complex with inhibitory G protein. MedComm..

[33] Venkatakrishnan AJ (2013). Molecular signatures of G-protein-coupled receptors. Nature.

[34] Soga T (2003). Molecular identification of nicotinic acid receptor. Biochem. Biophys. Res. Commun..

[35] Gharbaoui T (2007). Agonist lead identification for the high affinity niacin receptor GPR109a. Bioorg. Med. Chem. Lett..

[36] Boatman PD, Richman JG, Semple G (2008). Nicotinic acid receptor agonists. J. Med. Chem..

[37] Rodriguez A, Laio A (2014). Machine learning. Clustering by fast search and find of density peaks. Science.

[38] Kruse AC (2013). Activation and allosteric modulation of a muscarinic acetylcholine receptor. Nature.

[39] Shao Z (2022). Molecular insights into ligand recognition and activation of chemokine receptors CCR2 and CCR3. Cell Discov..

[40] Hua T (2017). Crystal structures of agonist-bound human cannabinoid receptor CB. Nature.

[41] Luginina A (2019). Structure-based mechanism of cysteinyl leukotriene receptor inhibition by antiasthmatic drugs. Sci. Adv..

[42] Zhang C (2012). High-resolution crystal structure of human protease-activated receptor 1. Nature.

[43] Kang Y (2018). Cryo-EM structure of human rhodopsin bound to an inhibitory G protein. Nature.

[44] Koehl A (2018). Structure of the µ-opioid receptor-G(i) protein complex. Nature.

[45] Krishna Kumar K (2019). Structure of a signaling cannabinoid receptor 1-G protein complex. Cell.

[46] Hanson J (2010). Nicotinic acid- and monomethyl fumarate-induced flushing involves GPR109A expressed by keratinocytes and COX-2-dependent prostanoid formation in mice. J. Clin. Invest..

[47] Richman JG (2007). Nicotinic acid receptor agonists differentially activate downstream effectors. J. Biol. Chem..

[48] Walters RW (2009). beta-Arrestin1 mediates nicotinic acid-induced flushing, but not its antilipolytic effect, in mice. J. Clin. Invest..

[49] Ahmed K, Tunaru S, Offermanns S (2009). GPR109A, GPR109B and GPR81, a family of hydroxy-carboxylic acid receptors. Trends Pharmacol. Sci..

[50] Ahmed K (2009). Deorphanization of GPR109B as a receptor for the beta-oxidation intermediate 3-OH-octanoic acid and its role in the regulation of lipolysis. J. Biol. Chem..

[51] Koehl A (2018). Structure of the µ-opioid receptor-Gi protein complex. Nature.

[52] Liu Z (2016). 2.9 Å resolution cryo-EM 3D reconstruction of close-packed virus particles. Structure.

[53] Zheng SQ (2017). MotionCor2: anisotropic correction of beam-induced motion for improved cryo-electron microscopy. Nat. Methods.

[54] Jiang W, Guo F, Liu Z (2012). A graph theory method for determination of cryo-EM image focuses. J. Struct. Biol..

[55] Punjani A, Rubinstein JL, Fleet DJ, Brubaker MA (2017). cryoSPARC: algorithms for rapid unsupervised cryo-EM structure determination. Nat. Methods.

[56] Zhuang Y (2020). Structure of formylpeptide receptor 2-Gi complex reveals insights into ligand recognition and signaling. Nat. Commun..

[57] Webb B, Sali A (2016). Comparative protein structure modeling using MODELLER. Curr. Protoc. Bioinformatics.

[58] Jo S, Kim T, Iyer VG, Im W (2008). CHARMM-GUI: a web-based graphical user interface for CHARMM. J. Comput. Chem..

[59] Lomize MA (2012). OPM database and PPM web server: resources for positioning of proteins in membranes. Nucleic Acids Res..

[60] Huang J (2017). CHARMM36m: an improved force field for folded and intrinsically disordered proteins. Nat. Methods.

[61] Vanommeslaeghe K (2010). CHARMM general force field: a force field for drug-like molecules compatible with the CHARMM all-atom additive biological force fields. J. Comput. Chem..

[62] Abraham MJ (2015). GROMACS: high performance molecular simulations through multi-level parallelism from laptops to supercomputers. SoftwareX.

[63] Berendsen H (1984). Molecular-dynamics with coupling to an external bath. J. Chem. Phys..

[64] Essmann U (1995). A smooth particle mesh Ewald method. J. Chem. Phys..

[65] Darden T, York D, Pedersen L (1993). Particle mesh Ewald: an Nlog(N) method for Ewald sums in large systems. J. Chem. Phys..

[66] McGibbon RT (2015). MDTraj: a modern open library for the analysis of molecular dynamics trajectories. Biophys. J..

[67] Valdés-Tresanco MS, Valdés-Tresanco ME, Valiente PA, Moreno E (2021). gmx_MMPBSA: a new tool to perform end-state free energy calculations with GROMACS. J. Chem. Theory Comput..

[68] Qiao A (2020). Structural basis of Gs and Gi recognition by the human glucagon receptor. Science.

[69] Hua, T. et al. Activation and signaling mechanism revealed by cannabinoid receptor-Gi complex structures. *Cell* **180**, 655–665.e18 (2020). 10.1016/j.cell.2020.01.008 PMC7898353 32004463

