# Supplementary material for: Structural insights into ligand recognition and selectivity of the human hydroxycarboxylic acid receptor HCAR2
Source: Cell Discov. 2023 Nov 28;9:118. doi: 10.1038/s41421-023-00610-7 (PMC10682194; doi:10.1038/s41421-023-00610-7)
Supplement: Supplementary file 1 — Supplementary materials [file 41421_2023_610_MOESM1_ESM.pdf]

Supplementary materials

Structural insights into ligand recognition and selectivity of the human hydroxycarboxylic acid receptor HCAR2

Xin Pan[1,2 †], Fang Ye[1 †], Peiruo Ning[1], Zhiyi Zhang[1], Xinyu Li[3], Binghao Zhang[1], Qian Wang[1], Geng Chen[1], Wei Gao[1], Chen Qiu[1], Zhangsong Wu[1], Jiancheng Li[4], Lizhe Zhu[3 *], Jiang Xia[5 *], Kaizheng Gong[2 *], Yang Du[1 *]

[1] Kobilka Institute of Innovative Drug Discovery, Shenzhen Key Laboratory of Steroid Drug Discovery and Development, School of Medicine, the Chinese University of Hong Kong, Shenzhen 518172, Guangdong, China

[2] Department of Cardiology, Central Laboratory, The Affiliated Hospital of Yangzhou University, Yangzhou University, Yangzhou 225000, Jiangsu, China

[3] Warshel Institute for Computational Biology, School of Medicine, the Chinese University of Hong Kong, Shenzhen 518172, Guangdong, China

[4] Instrumental Analysis Center, Shenzhen University, Shenzhen 518060, Guangdong, China

[5] Department of Chemistry, the Chinese University of Hong Kong, Shatin, Hong Kong SAR, China

[†]These authors contributed equally to this article

***To whom correspondence should be addressed:**

Professor Yang Du

Email: yangdu@cuhk.edu.cn

Professor Kaizheng Gong

Email: yungkzh@163.com

Professor Jiang Xia

Email: jiangxia@cuhk.edu.hk

Professor Lizhe Zhu

Email: zhulizhe@cuhk.edu.cn

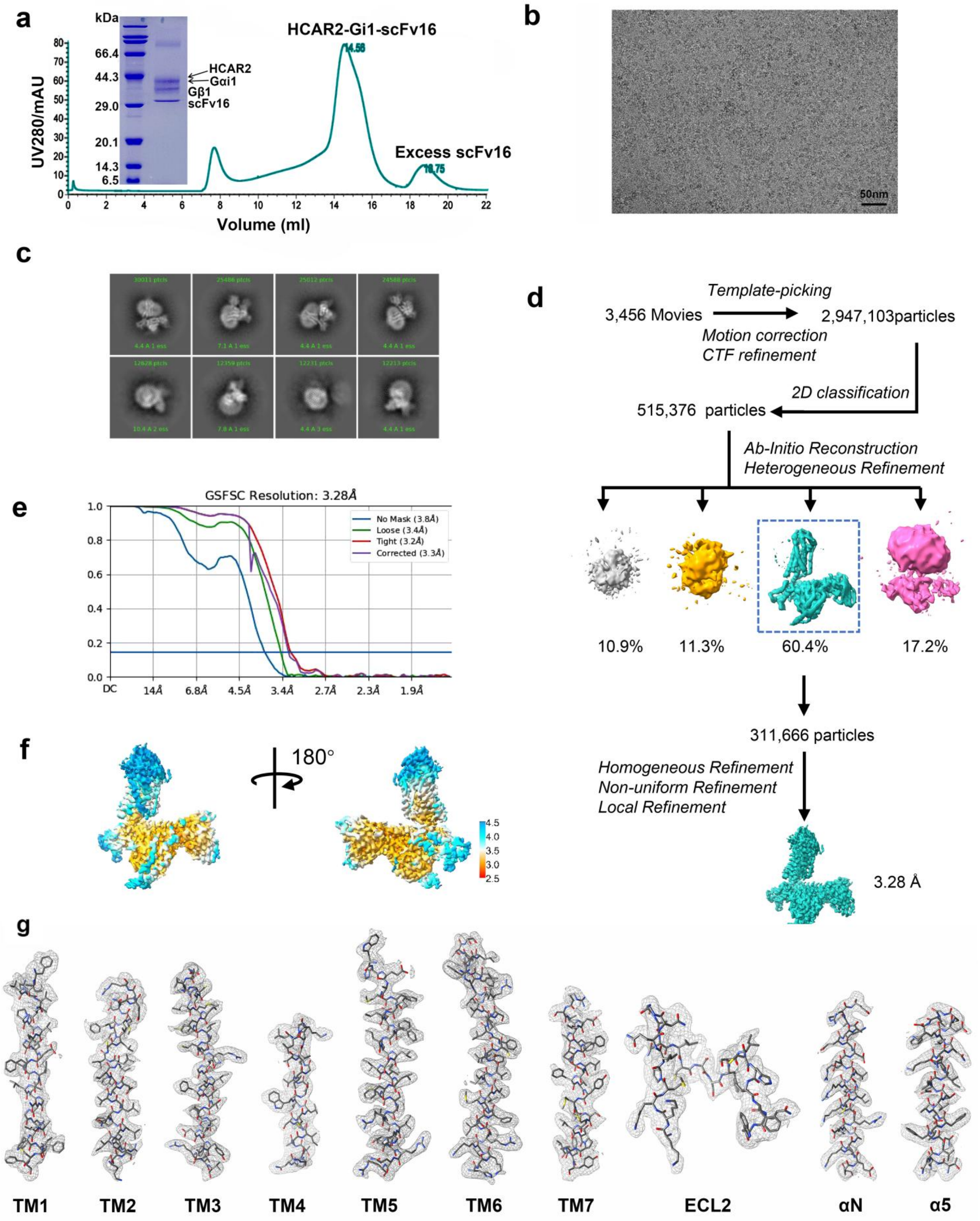

Supplementary Fig. S1 Cryo-EM data processing of the HCAR2-Gi1 signaling complex in the apo form. a Size exclusion chromatography profile and SDS-PAGE of the HCAR2-Gi1 complex. **b** Representative micrograph of the complex particles. **c** Representative 2D averages. **d** Workflow for cryo-EM image processing. **e** Gold-standard FSC curves of the 3D reconstructions. **f** Local resolution map of the complex. **g** Representative density maps and models for TM1–7 and ECL2 of HCAR2 and the α helices of Gαi1 (αN and α5). The density maps are shown at a contour level of 3.6 rmsd.

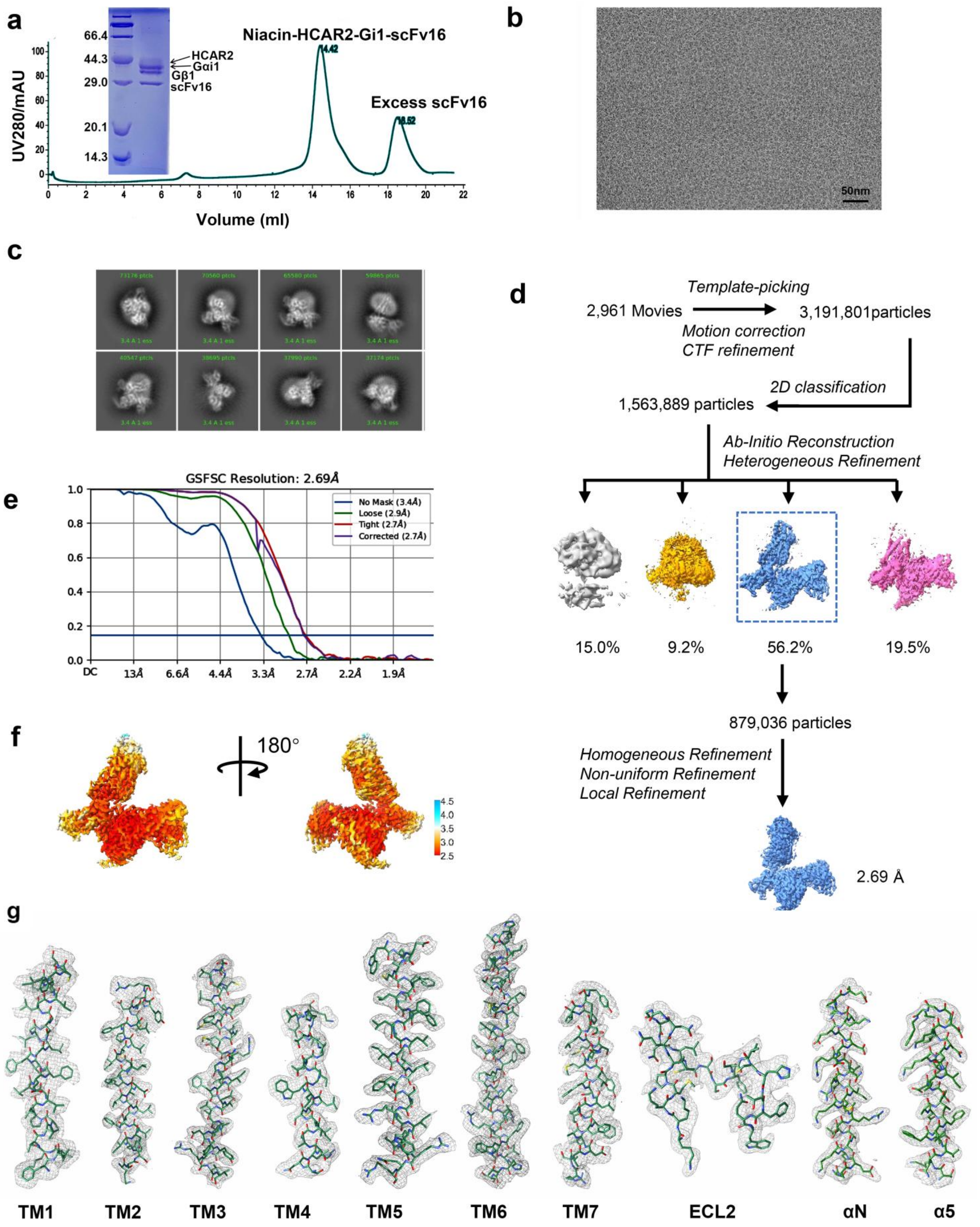

Supplementary Fig. S2 Cryo-EM data processing of the HCAR2-Gi1 signaling complex in the niacin-bound form. **a** Size exclusion chromatography profile and SDS-PAGE of the HCAR2-Gi1 complex bound with niacin. **b** Representative micrograph of the complex particles. **c** Representative 2D averages. **d** Workflow for cryo-EM image processing. **e** Gold-standard FSC curves of the 3D reconstructions. **f** Local resolution map of the complex. **g** Representative density maps and models for TM1–7 and ECL2 of HCAR2 and the α helices of Gαi1 (αN and α5). The density maps are shown at a contour level of 3.6 rmsd.

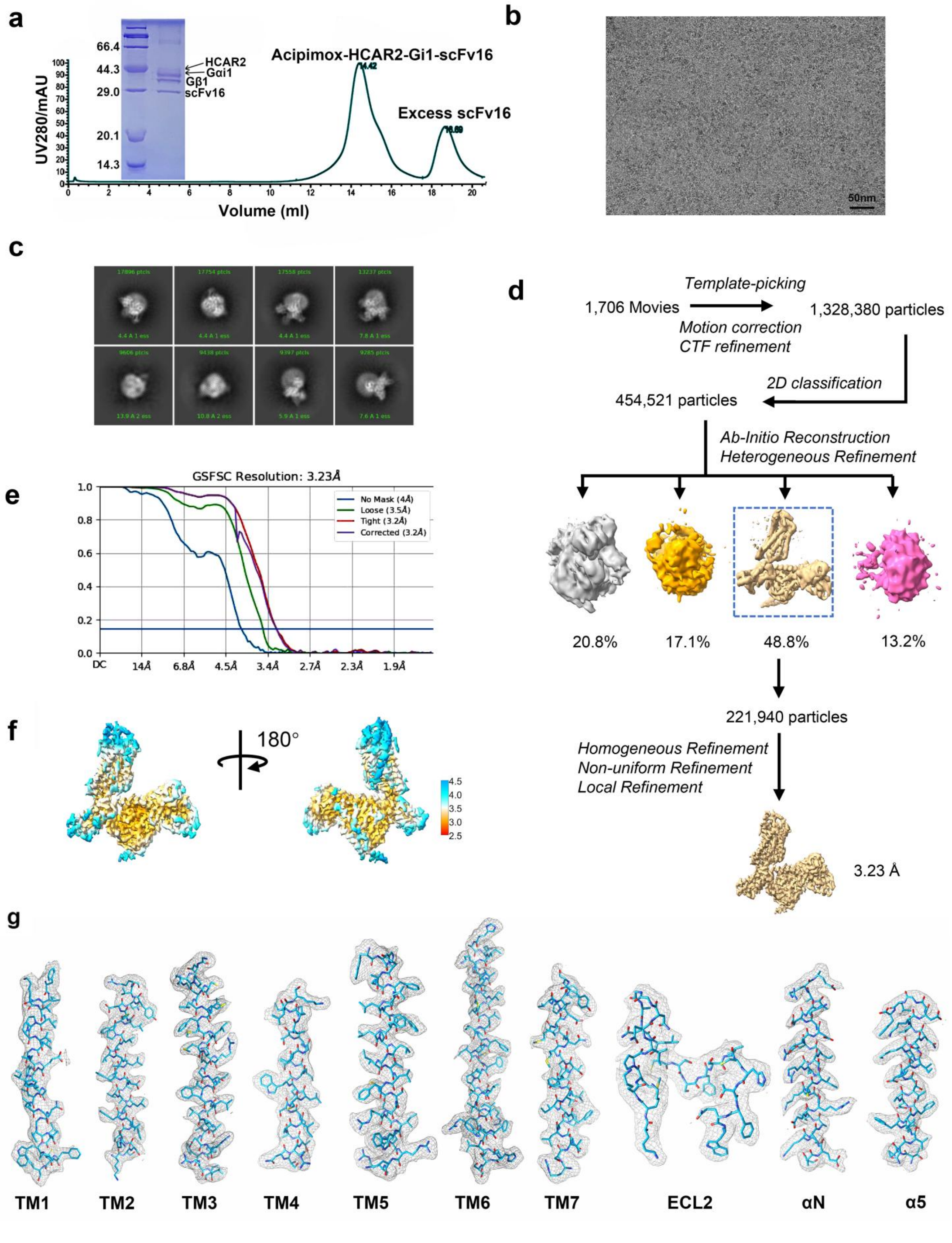

Supplementary Fig. S3 Cryo-EM data processing of the HCAR2-Gi1 signaling complex in the acipimox-bound form. **a** Size exclusion chromatography profile and SDS-PAGE of the HCAR2-Gi1 complex bound with acipimox. **b** Representative micrograph of the complex particles. **c** Representative 2D averages. **d** Workflow for cryo-EM image processing. **e** Gold-standard FSC curves of the 3D reconstructions. **f** Local resolution map of the complex. **g** Representative density maps and models for TM1–7 and ECL2 of HCAR2 and the α helices of Gαi1 (αN and α5). The density maps are shown at a contour level of 3.6 rmsd.

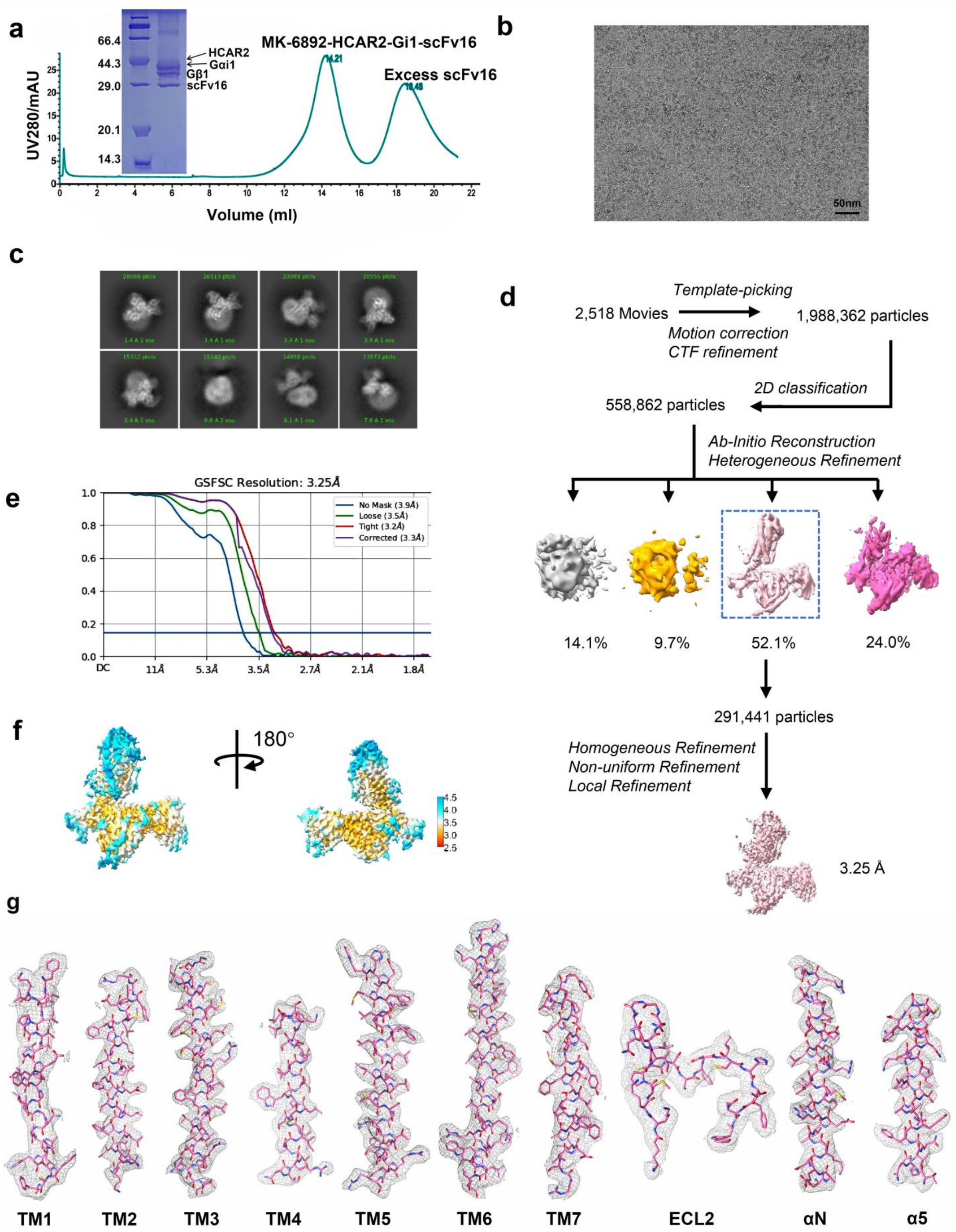

Supplementary Fig. S4 Cryo-EM data processing of the HCAR2-Gi1 signaling complex in the MK-6892-bound form. **a** Size exclusion chromatography profile and SDS-PAGE of the HCAR2-Gi1 complex bound with MK-6892. **b** Representative micrograph of the complex particles. **c** Representative 2D averages. **d** Workflow for cryo-EM image processing. **e** Gold-standard FSC curves of the 3D reconstructions. **f** Local resolution map of the complex. **g** Representative density maps and models for TM1–7 and ECL2 of HCAR2 and the α helices of Gαi1 (αN and α5). The density maps are shown at a contour level of 3.6 rmsd.

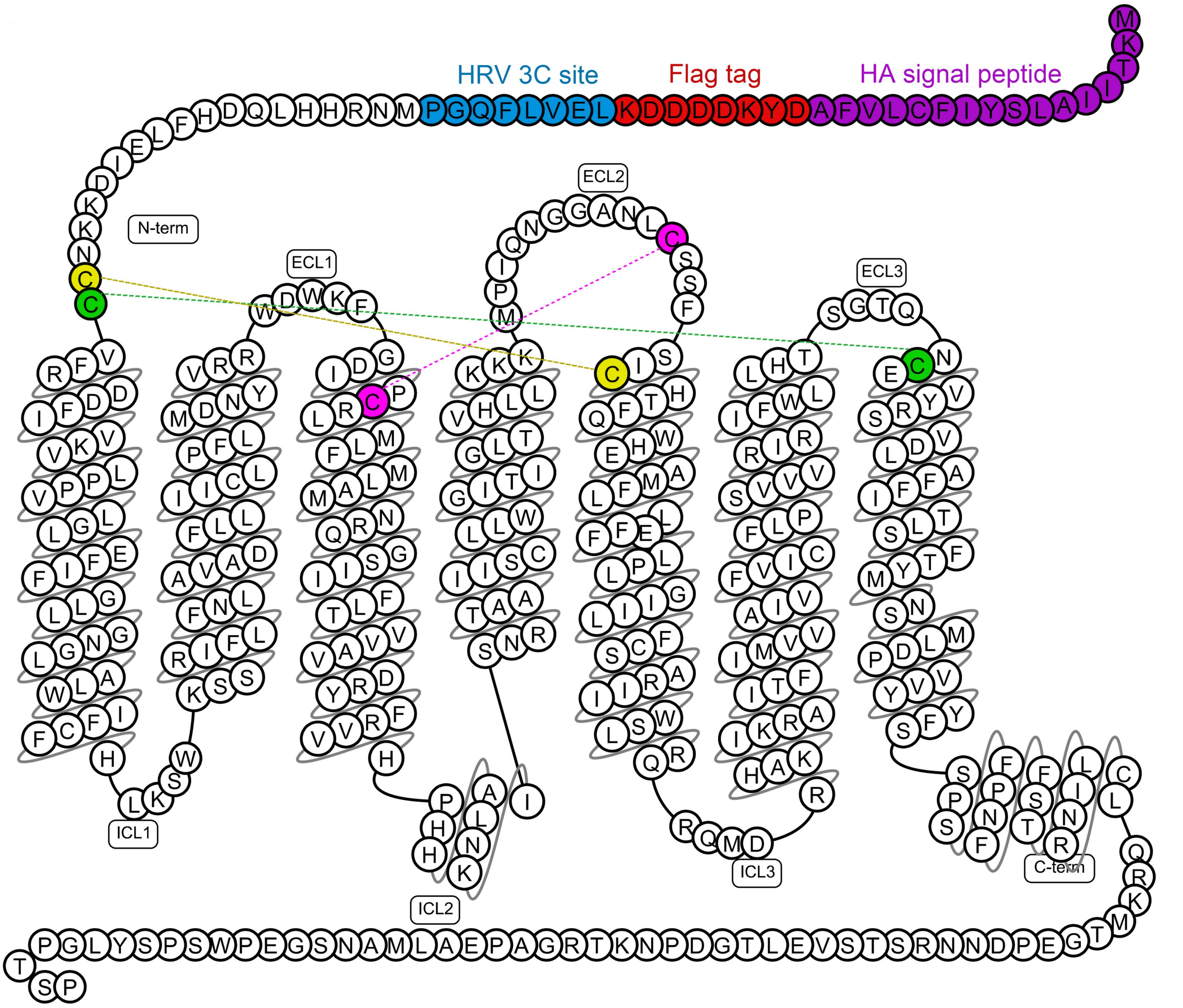

Supplementary Fig. S5 Schematic diagram of the human HCAR2 construct used in this study. HA signal peptide (mauve), Flag epitope (red), and HRV 3C site (dark blue) are added in the N terminus. Three disulfide bonds are connected as shown by dashed lines and colored in yellow, green, and fuchsia, respectively.

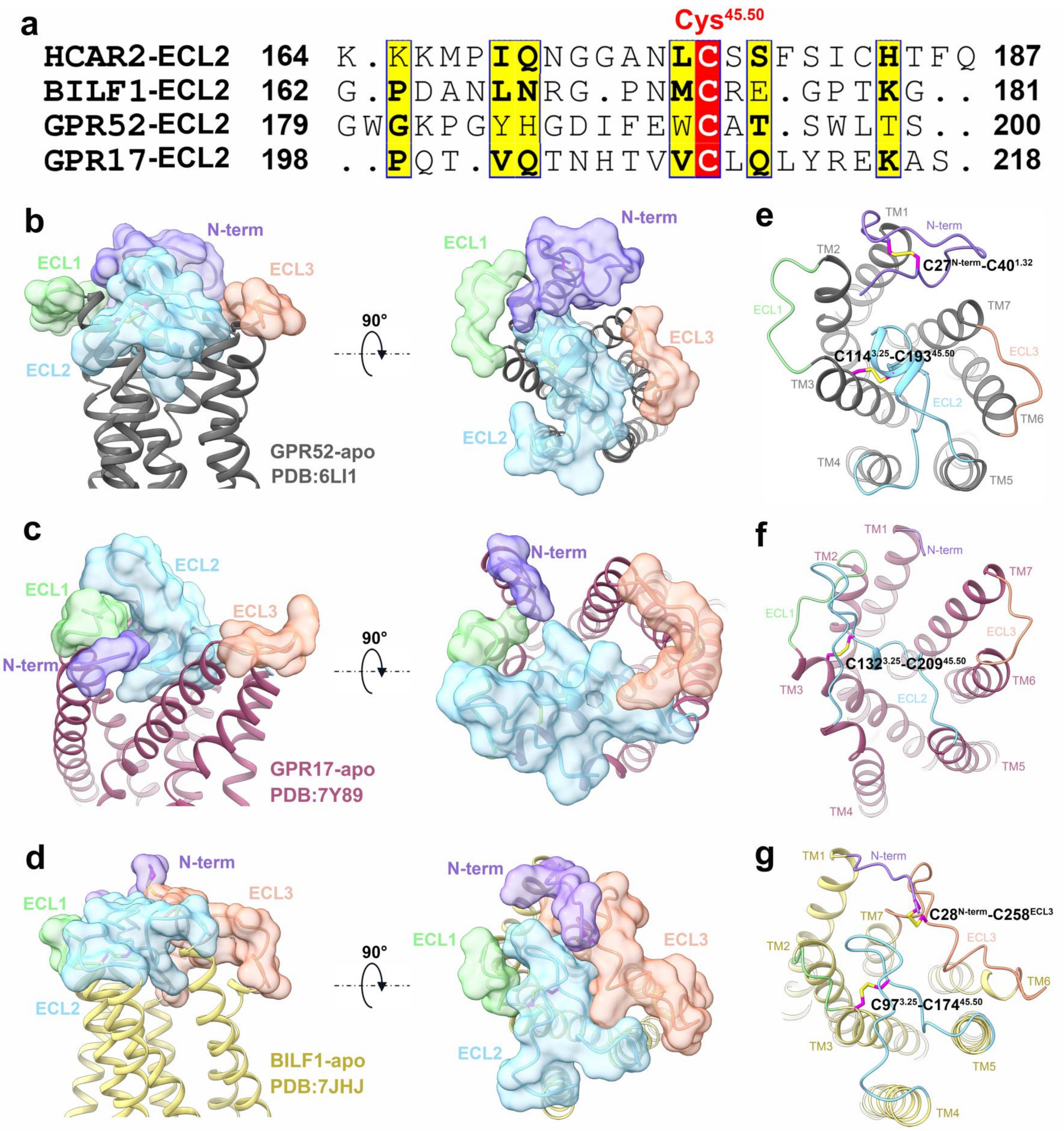

Supplementary Fig. S6 Sequence and structure of the reported GPCRs with self-activation.

a ECL2 sequence alignment of HCAR2 with GPR52, GPR17, and BILF1. The only conserved residue Cys$^{45.50}$ is colored red. **b–d** Extracellular architecture of GPR52 (PDB: 6LI1, dark gray), GPR17 (PDB: 7Y89, violet red), and BILF1 (PDB: 7JHJ, goldenrod yellow) from side and top views. The N-terminal loop (blue purple), ECL1 (light green), ECL2 (sky blue), and ECL3 (coral) are shown as transparent surface representations. **e–g** Disulfide bonds (magenta sticks) in the extracellular region of GPR52 (dark gray), GPR17 (violet red), and BILF1 (goldenrod yellow). The N-terminal loop (blue purple), ECL1 (light green), ECL2 (sky blue), and ECL3 (coral) are shown as cartoon model.

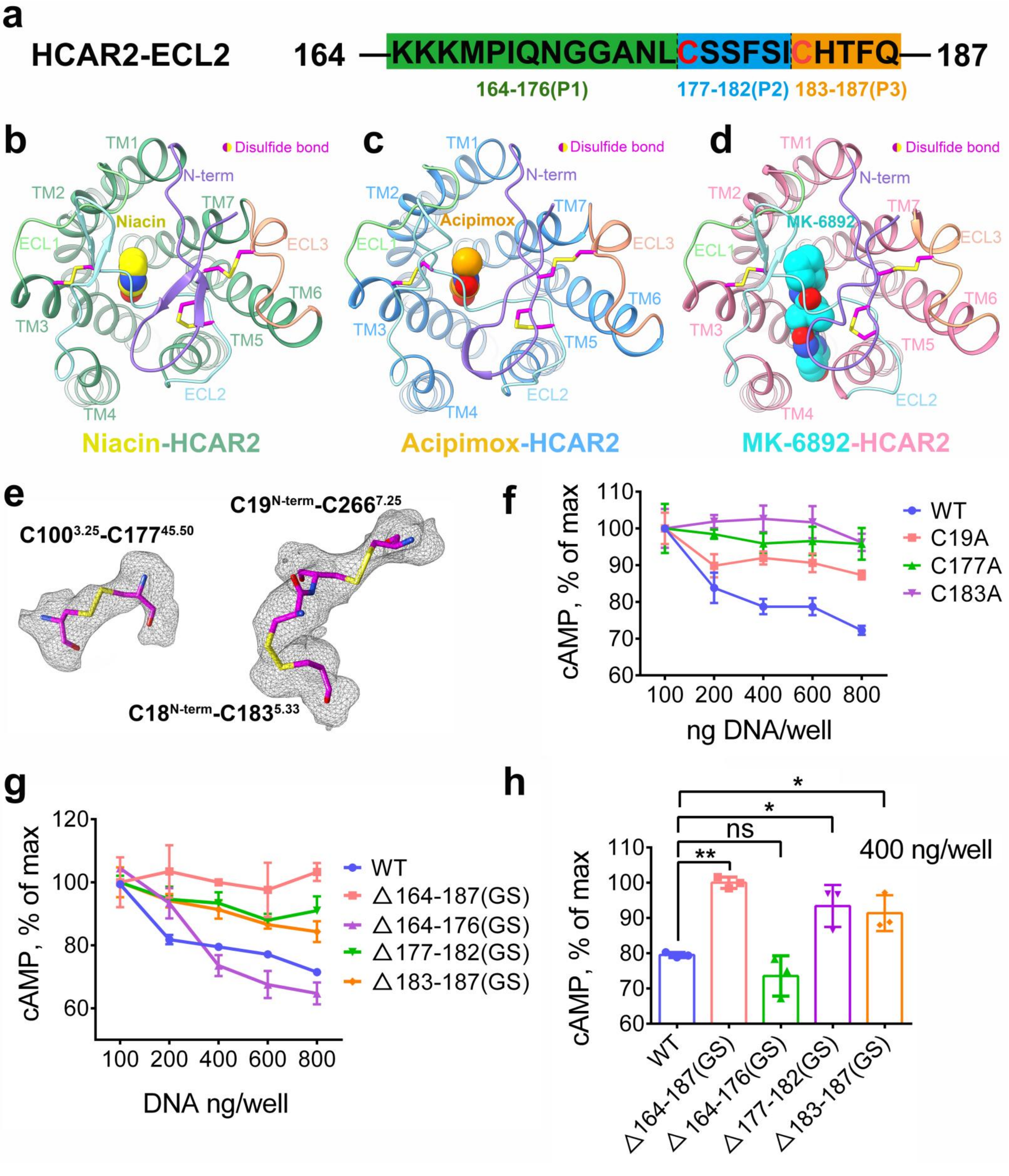

Supplementary Fig. S7 Effects of disulfide bonds on the structural stability of ECL2.

a ECL2 is divided into P1 segment (K164–L176), P2 segment (C177–I182), and P3 segment (C183–Q187). The extracellular conformations of niacin- **(b)**, acipimox- **(c)**, and MK-6892-bound **(d)** HCAR2 complexes are stabilized by three disulfide bonds. The structures of HCAR2 and agonists are colored differently. Forest green, niacin-HCAR2; deep sky blue, acipimox-HCAR2; hot pink, MK-6892-HCAR2; blue purple, N-terminal loop; light green, ECL1; sky blue, ECL2; coral, ECL3;

yellow, niacin; dark orange, acipimox; cyan, MK-6892; magenta sticks, disulfide bonds. **e** Density maps of disulfide bonds in the niacin-bound HCAR2 complex. The density maps are shown at a contour level of 3.6 rmsd. **f** Effects on Gi-mediated cAMP by single-point mutations of $C19^{N\text{-}term}$, $C177^{45.50}$, and $C183^{5.33}$. **g, h** Effects on Gi-mediated cAMP by replacing the ECL2 region, P1 segment, P2 segment, and P3 segment with a six-residue linker (GGSGGS), respectively. The data are presented as mean ± SEM, one-way analysis of variance (ANOVA), *$p < 0.05$, **$p < 0.01$, ***$p < 0.001$. The experiments were performed in triplicate.

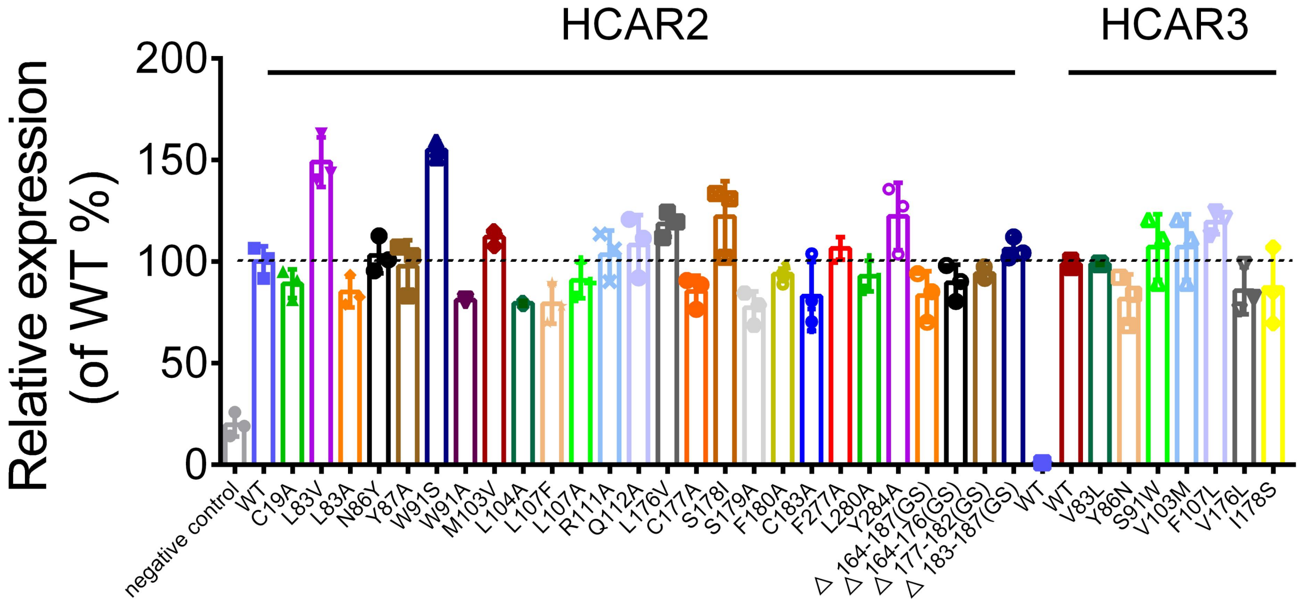

Supplementary Fig. S8 Relative expression of wild-type and mutants of HCAR2 and HCAR3. Relative cellular expression is determined by FACS analysis. The data are presented as means ± SEM. The experiments were performed in triplicates.

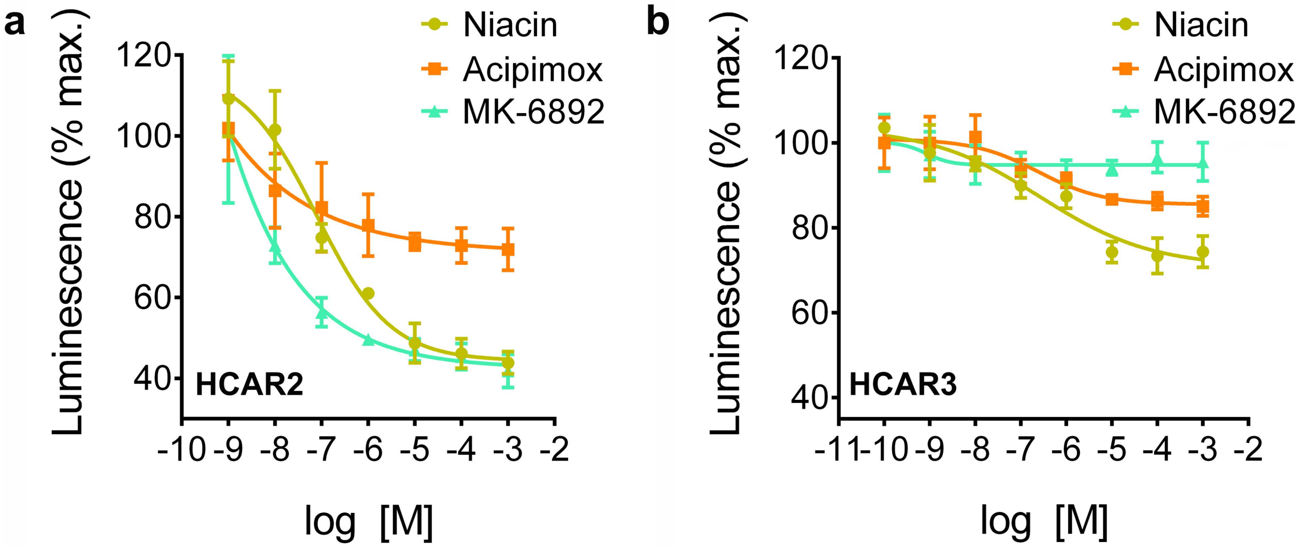

Supplementary Fig. S9 Coupling of HCAR2 (a) and HCAR3 (b) with Gi1 protein activated by niacin, acipimox, and MK-6892.

The data are presented as mean ± SEM. The experiments were performed in triplicate.

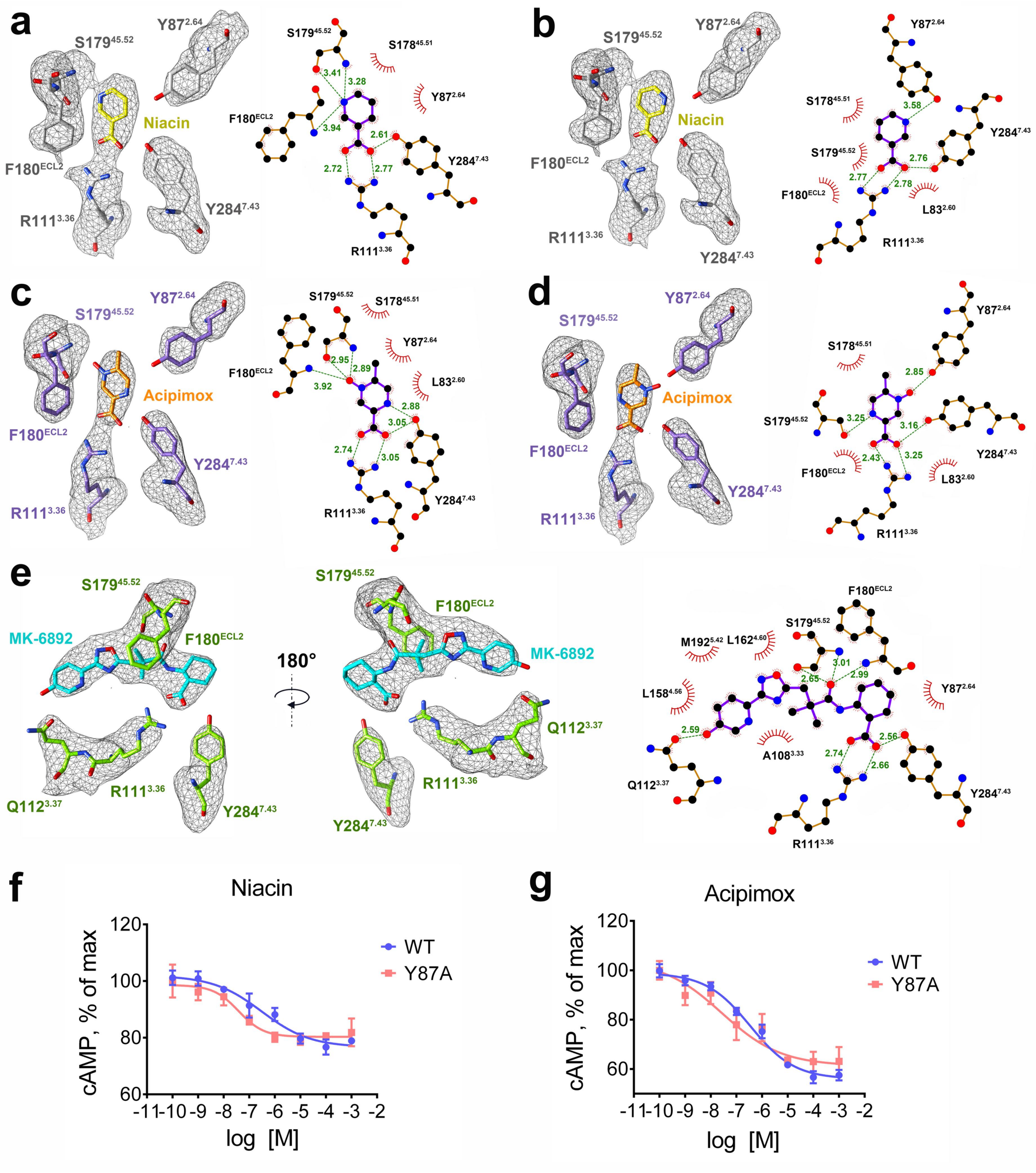

Supplementary Fig. S10 Determining the binding modes of niacin, acipimox, and MK-6892 in the ligand-binding pocket of HCAR2.

Density maps and 2D interactions of niacin (yellow) with the surrounding residues when the pyridinic-N atom of niacin is oriented in the S179$^{45.52}$ **(a)** or Y87$^{2.64}$ **(b)** orientation. Density maps and 2D interactions of acipimox (dark orange) with the surrounding residues when the oxide moiety of acipimox is oriented in the S179$^{45.52}$ **(c)** or Y87$^{2.64}$ **(d)** orientation. **e** Density maps and 2D interactions of MK-6892 (cyan) with the surrounding residues. The density maps are shown at a contour level of 3.6 rmsd. **f, g** Effect on Gi-mediated cAMP by single-point mutation of Y87$^{2.64}$A. The data are presented as mean ± SEM. The experiments were performed in triplicate.

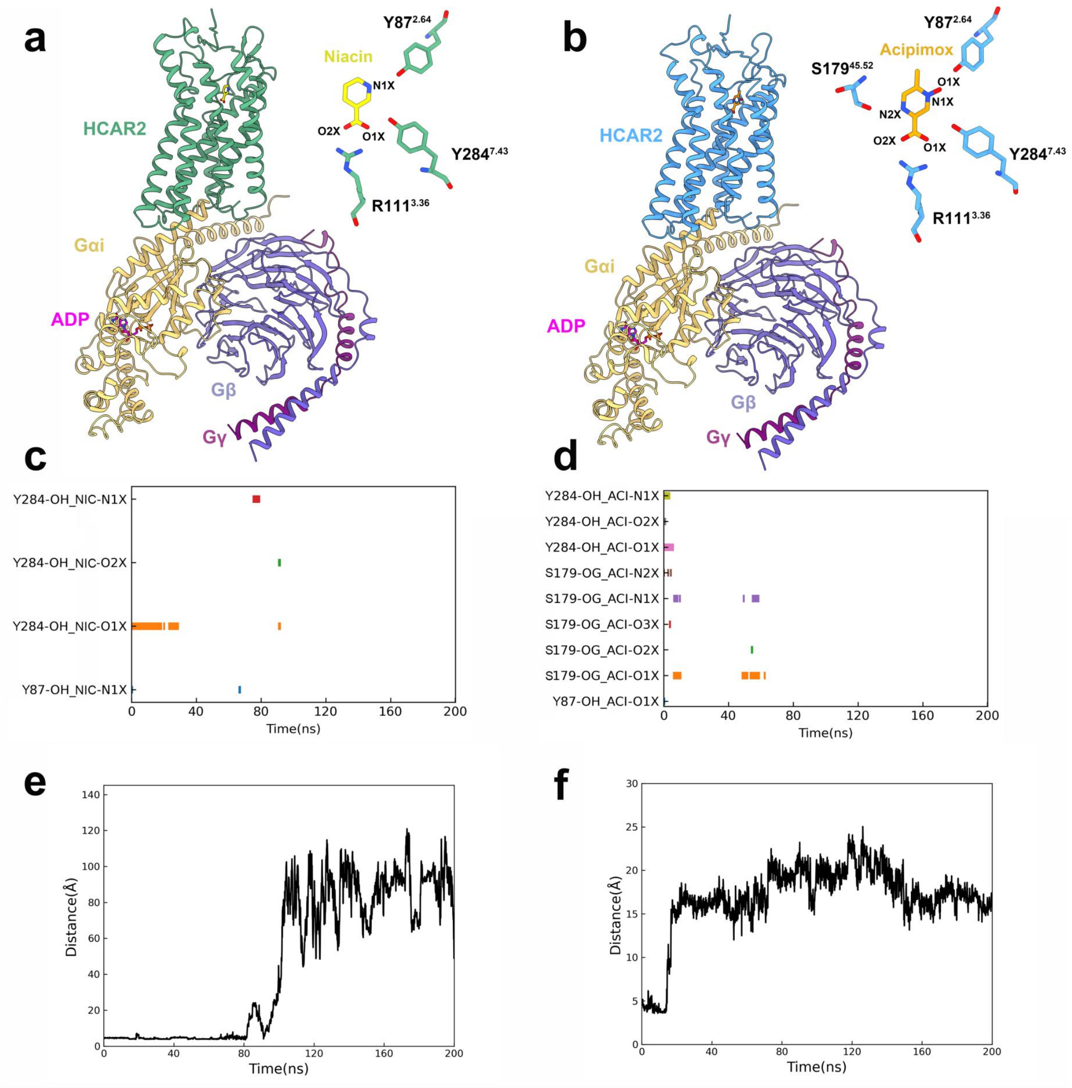

Supplementary Fig. S11 Molecular dynamics simulations of the niacin- and acipimox-bound HCAR2 toward the Y87$^{2.64}$ orientation.

a, b Starting structures of niacin- and acipimox-bound HCAR2 toward the Y87$^{2.64}$ orientation for simulation. Missing part of Gαi is completed by the MODELLER. Hydrogen bond lifetime analysis for Y284$^{7.43}$, S179$^{45.52}$ and Y87$^{2.64}$ with ligand in niacin-HCAR2 system (**c**), acipimox-HCAR2 system (**d**). Salt bridge lifetime analysis between R111$^{3.36}$ and ligand in niacin-HCAR2 system (**e**), and acipimox-HCAR2 system (**f**). Forest green, niacin-HCAR2 toward the Y87$^{2.64}$ orientation; deep sky blue, acipimox-HCAR2 toward the Y87$^{2.64}$ orientation; yellow, niacin; dark orange, acipimox; magenta sticks, ADP.

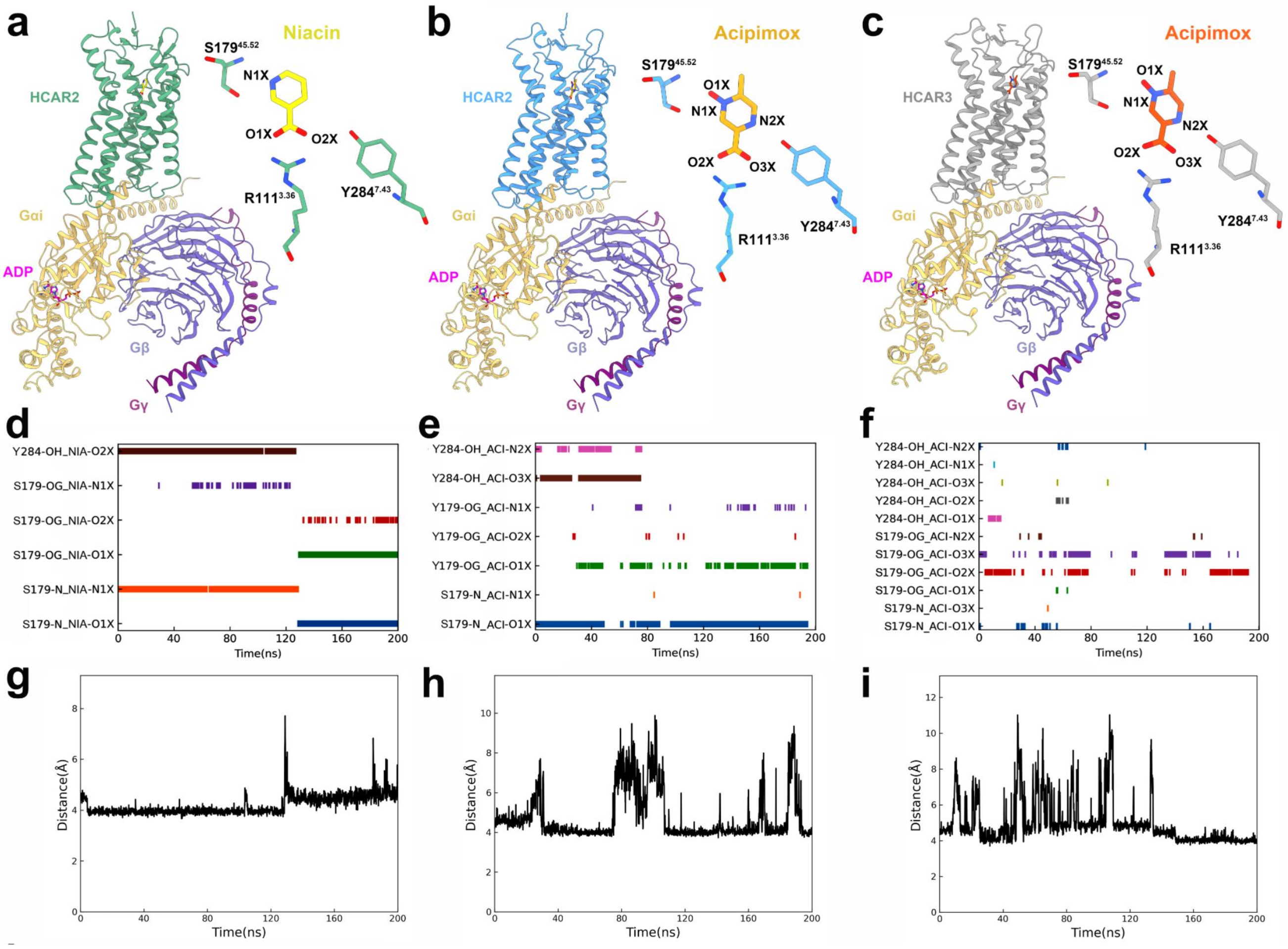

Supplementary Fig. S12 Molecular dynamics simulations of the niacin- and acipimox-bound HCAR2 toward the S179$^{45.52}$ orientation as well as the predicted acipimox-bound HCAR3 complex.

a−c Starting structures of niacin- and acipimox-bound HCAR2, and acipimox-bound HCAR3 for simulation. Missing part of Gαi is completed by the MODELLER. Hydrogen bond lifetime analysis for Y284$^{7.43}$ and S179$^{45.52}$ with ligand in niacin-HCAR2 system (**d**), acipimox-HCAR2 system (**e**), and acipimox-HCAR3 system (**f**). Salt bridge lifetime analysis between R111$^{3.36}$ and ligand in niacin-HCAR2 system (**g**), acipimox-HCAR2 system (**h**), and acipimox-HCAR3 system (**i**). Forest green, niacin-HCAR2; deep sky blue, acipimox-HCAR2; gray, HCAR3; yellow, niacin; dark orange, acipimox in HCAR2; orange red, acipimox in HCAR3; magenta sticks, ADP.

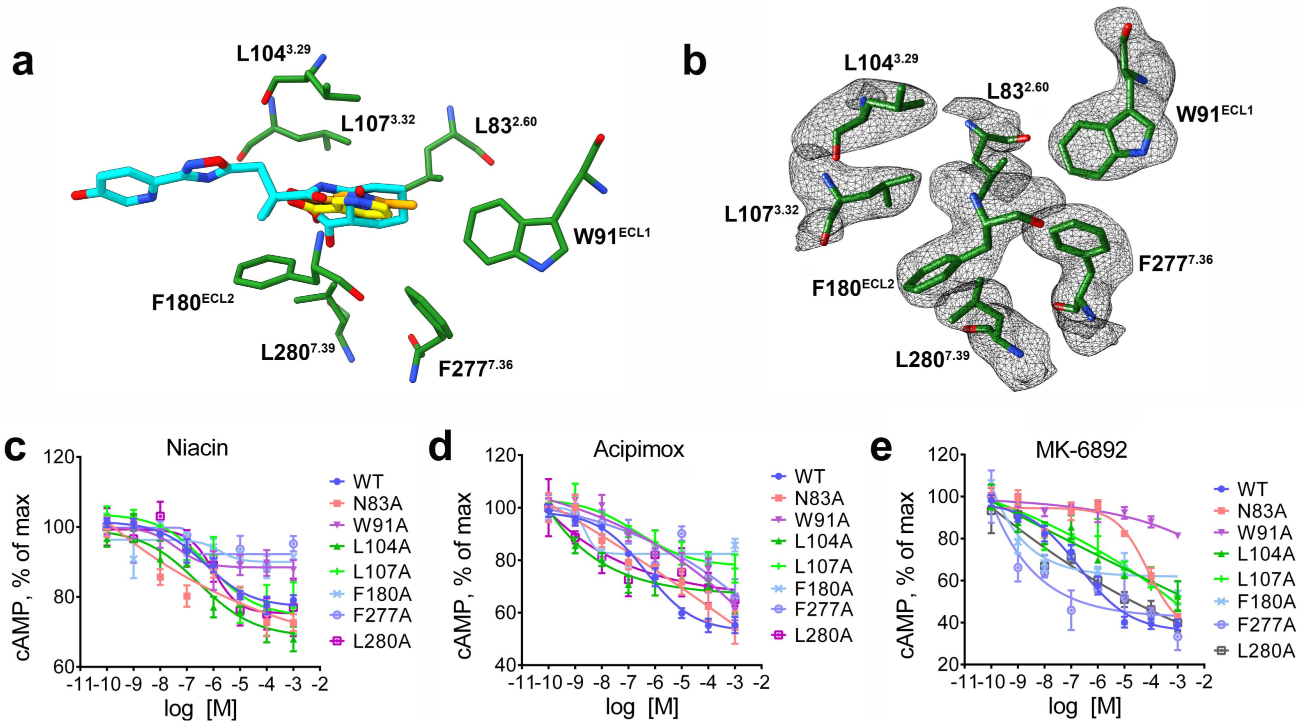

Supplementary Fig. S13 Effects of hydrophobic interactions on HCAR2 activation.

a Hydrophobic residues (green) around the niacin (yellow), acipimox (dark orange), and MK-6892 (cyan) in the orthosteric pocket. **b** Density maps of the hydrophobic residues in the orthosteric pocket. The density maps are shown at a contour level of 3.6 rmsd. **c–e** Mutations of the hydrophobic residues influence the agonistic activity of niacin, acipimox, and MK-6892 to varying degrees. The data are presented as mean ± SEM. The experiments were performed in triplicate.

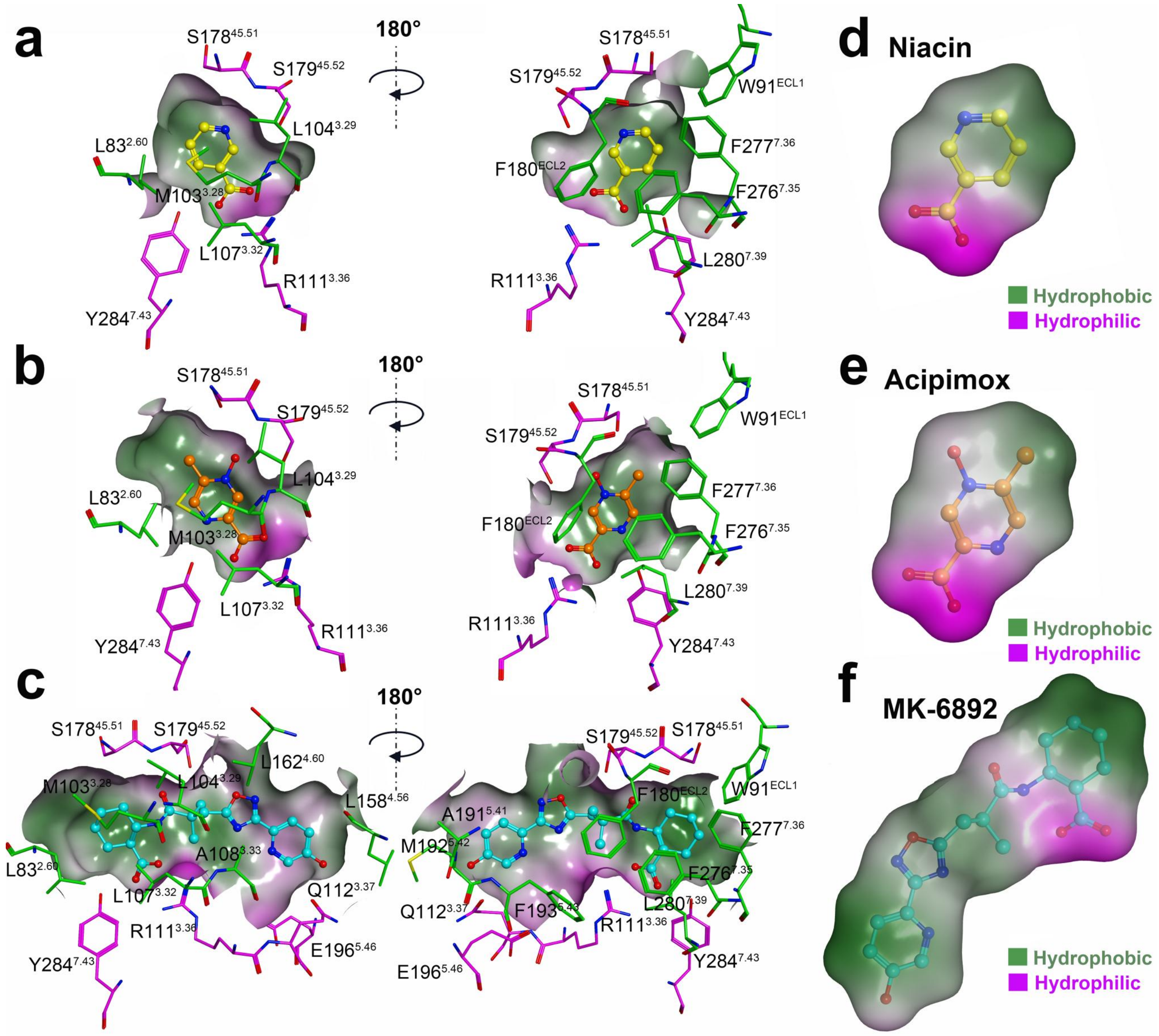

Supplementary Fig. S14 Analysis of the hydrophilic, hydrophobic, and charged properties of niacin, acipimox, MK-6892, and orthosteric pocket.

Key polar and hydrophobic residues in the orthosteric pockets of niacin **(a)**, acipimox **(b)**, and MK-6892 **(c)** complexes. The hydrophilic, hydrophobic, and charged properties of niacin **(d)**, acipimox **(e)**, and MK-6892 **(f)**. The pockets and ligands are shown as surfaces and colored according to hydrophobicity (green) and hydrophilicity (magenta).

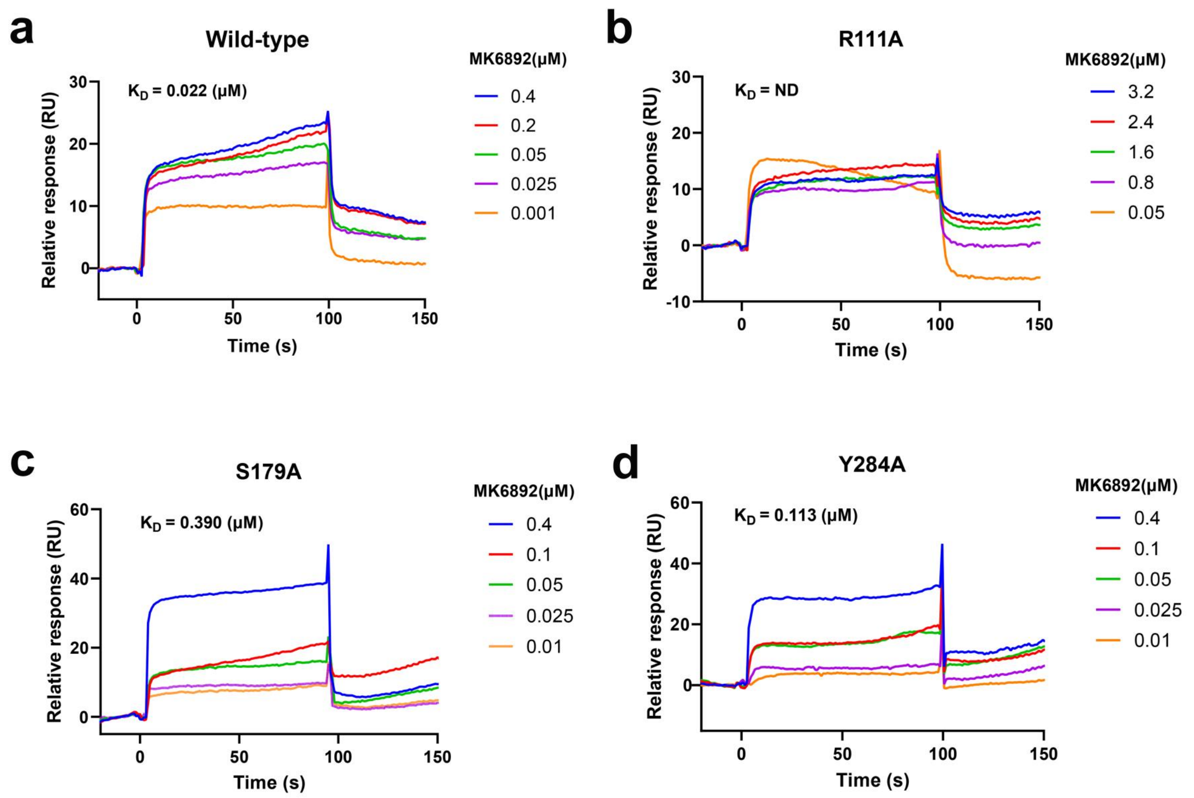

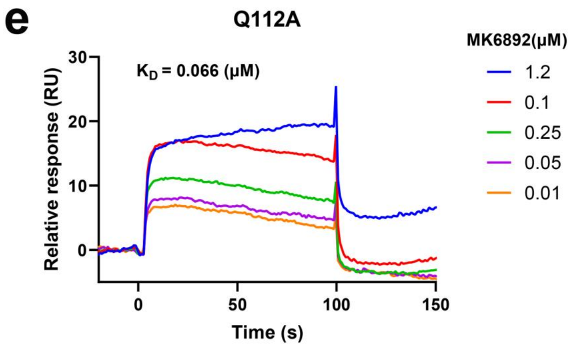

f Affinity parameters

	MK-6892 K_D (μM)	Niacin K_D (μM)	Acipimox K_D (μM)
Wild-type	0.022	0.058	0.429
R111A	ND	ND	ND
S179A	0.39	0.432	4.003
Y284A	0.113	0.415	3.027
Q112A	0.066	-	-

Supplementary Fig. S15 Binding affinity of the wild-type HCAR2 and its mutants for niacin, acipimox, and MK-6892 measured by SPR.

The SPR results of the wild-type HCAR2 **(a)** and its mutants R111$^{3.36}$A **(b)**, S179$^{45.52}$A **(c)**, Y284$^{7.43}$A **(d)**, and Q112$^{3.37}$A **(e)** for MK-6892. **f** Binding affinity parameters of wild-type HCAR2 and its mutants for all three agonists.

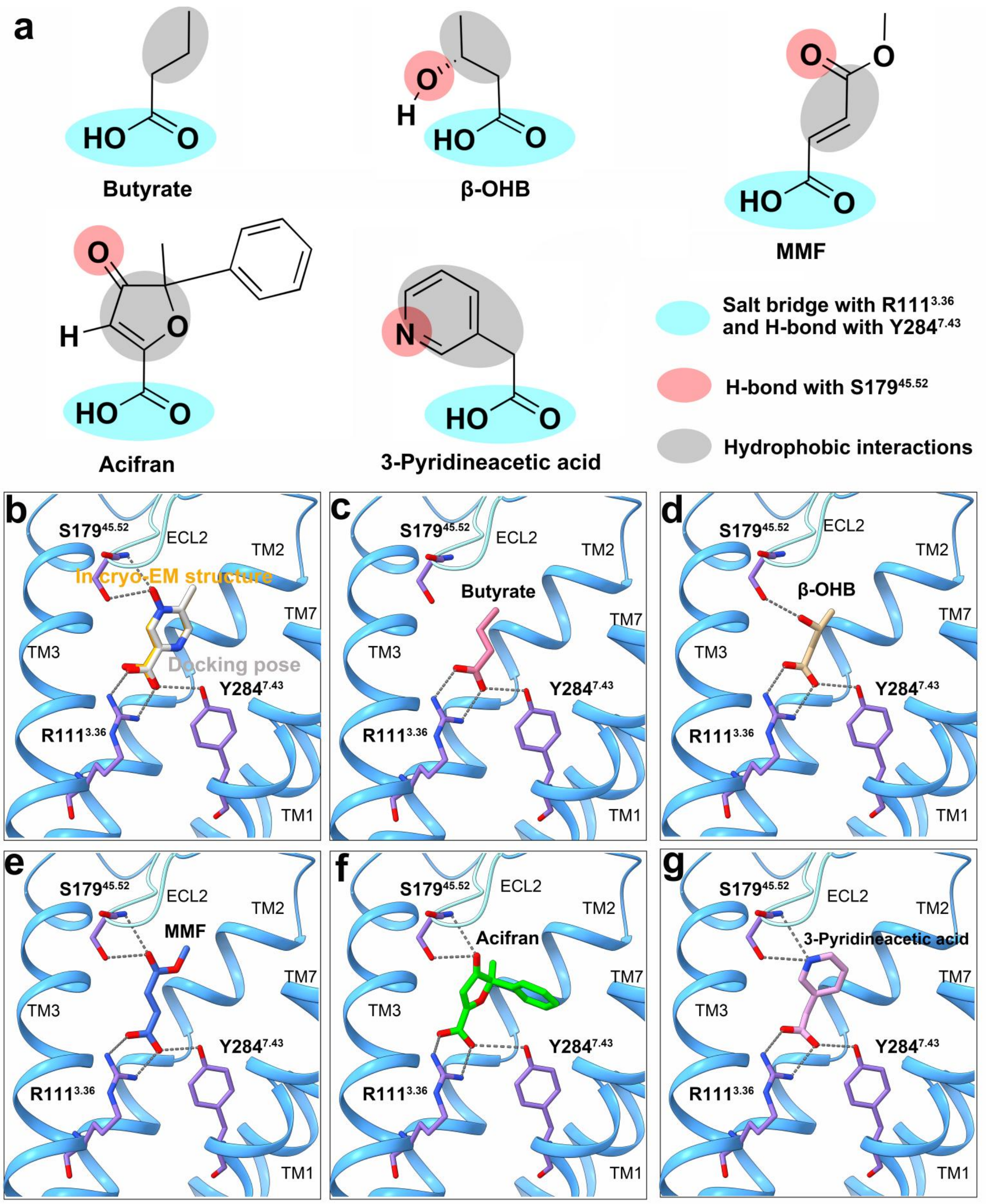

Supplementary Fig. S16 Predicted interactions of representative agonists with HCAR2.

a Chemical structures of butyrate, β-OHB, MMF, acifran, and 3-pyridineacetic acid. **b** Superposition of the acipimox cryo-EM structure (dark orange) and docking pose (silver). **c–g** Predicted binding modes of butyrate, β-OHB, MMF, acifran, and 3-pyridineacetic acid. The polar interactions are indicated by dark gray dashed lines. The structures of HCAR2 and agonists are colored differently. Deep sky blue, acipimox -HCAR2; light sky blue, ECL2; medium purple sticks, residues formed polar interactions; pale violet red, butyrate; tan, β-OHB; royal blue, MMF; green, acifran; plum, 3-pyridineacetic acid.

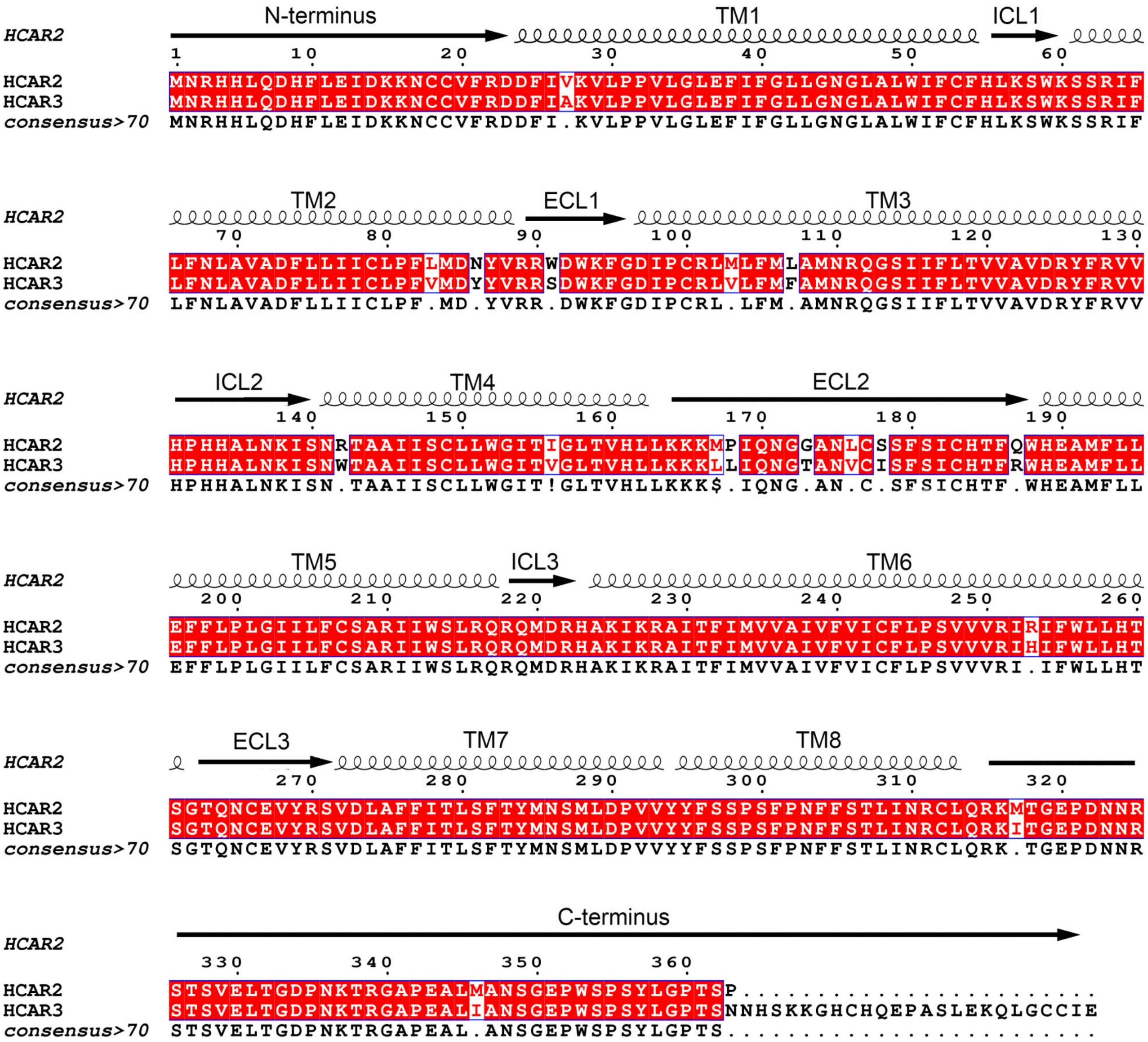

Supplementary Fig. S17 Sequence alignment of HCAR2 and HCAR3 receptors.

Positions that are identical between the receptors are highlighted with a red background.

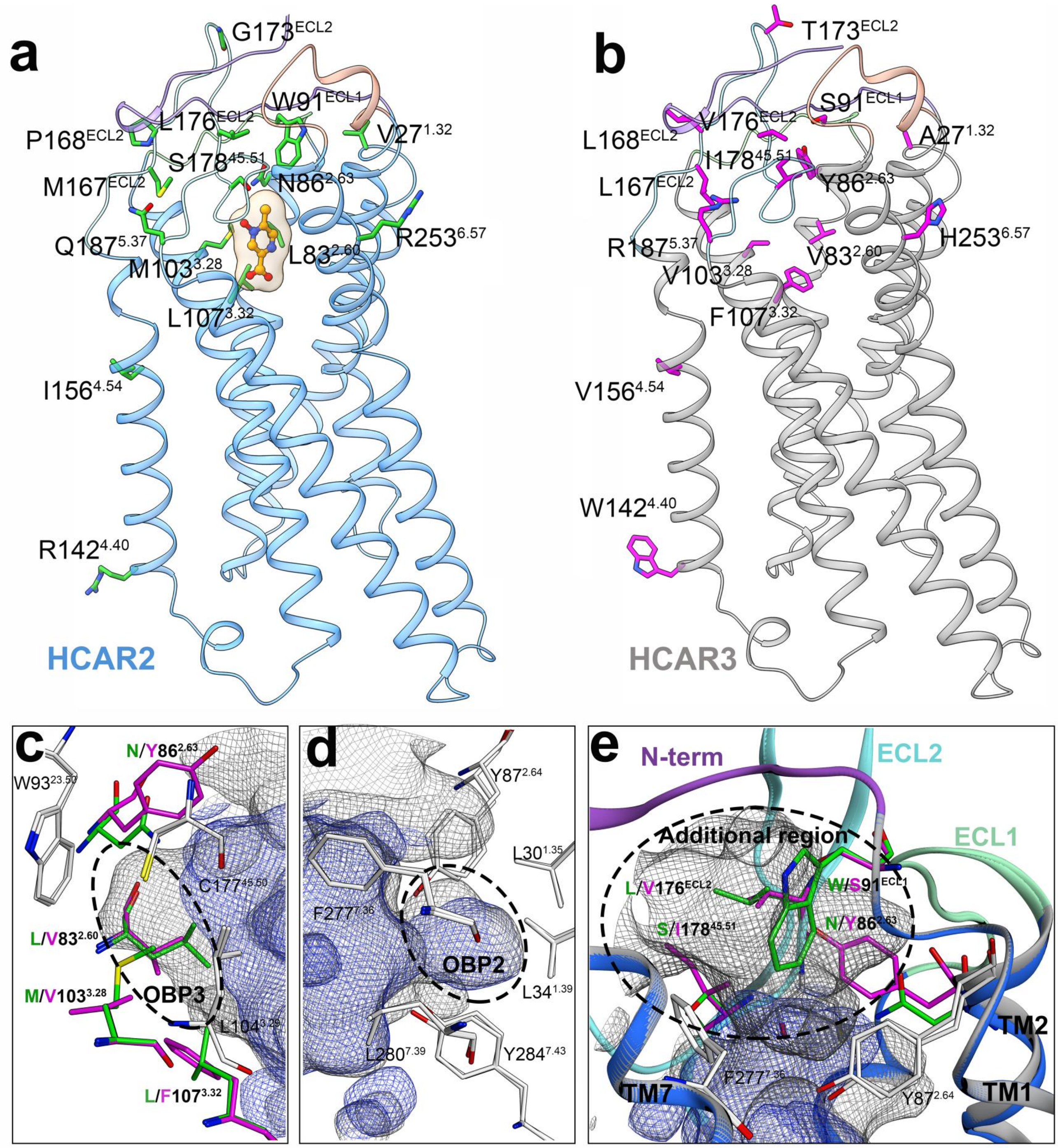

Supplementary Fig. S18 Comparison of the structural differences between HCAR2 and HCAR3 receptors.

Fine mapping of the 15 different residues in the HCAR2 **(a)** and HCAR3 **(b)** structures. **c−e** Orthosteric binding pockets of HCAR2 and HCAR3 are overlaid. The N-terminal loop (blue purple), ECL1 (light green), ECL2 (sky blue), and ECL3 (coral) in HCAR2 (deep sky blue) and HCAR3 (gray) are shown in cartoon representation. The 15 different residues in the HCAR2 (green), HCAR3 (magenta), and acipimox (dark orange) are shown in stick representation.

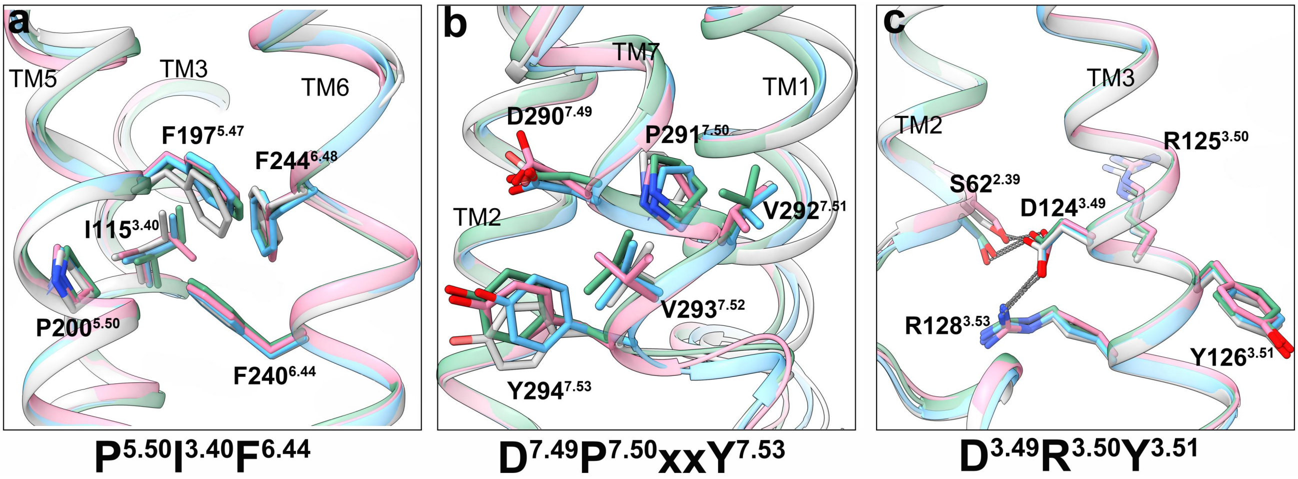

Supplementary Fig. S19 Conserved activation-related motifs of HCAR2 in four active states. Superposition of the $P^{5.50}$-$I^{3.40}$-$F^{6.44}$ **(a)**, $D^{7.49}P^{7.50}xxY^{7.53}$ **(b)**, and $D^{3.49}R^{3.50}Y^{3.51}$ **(c)** motifs. Light gray, apo-HCAR2; forest green, niacin-HCAR2; deep sky blue, acipimox-HCAR2; hot pink, MK-6892-HCAR2; dark gray dashed lines, polar interactions.

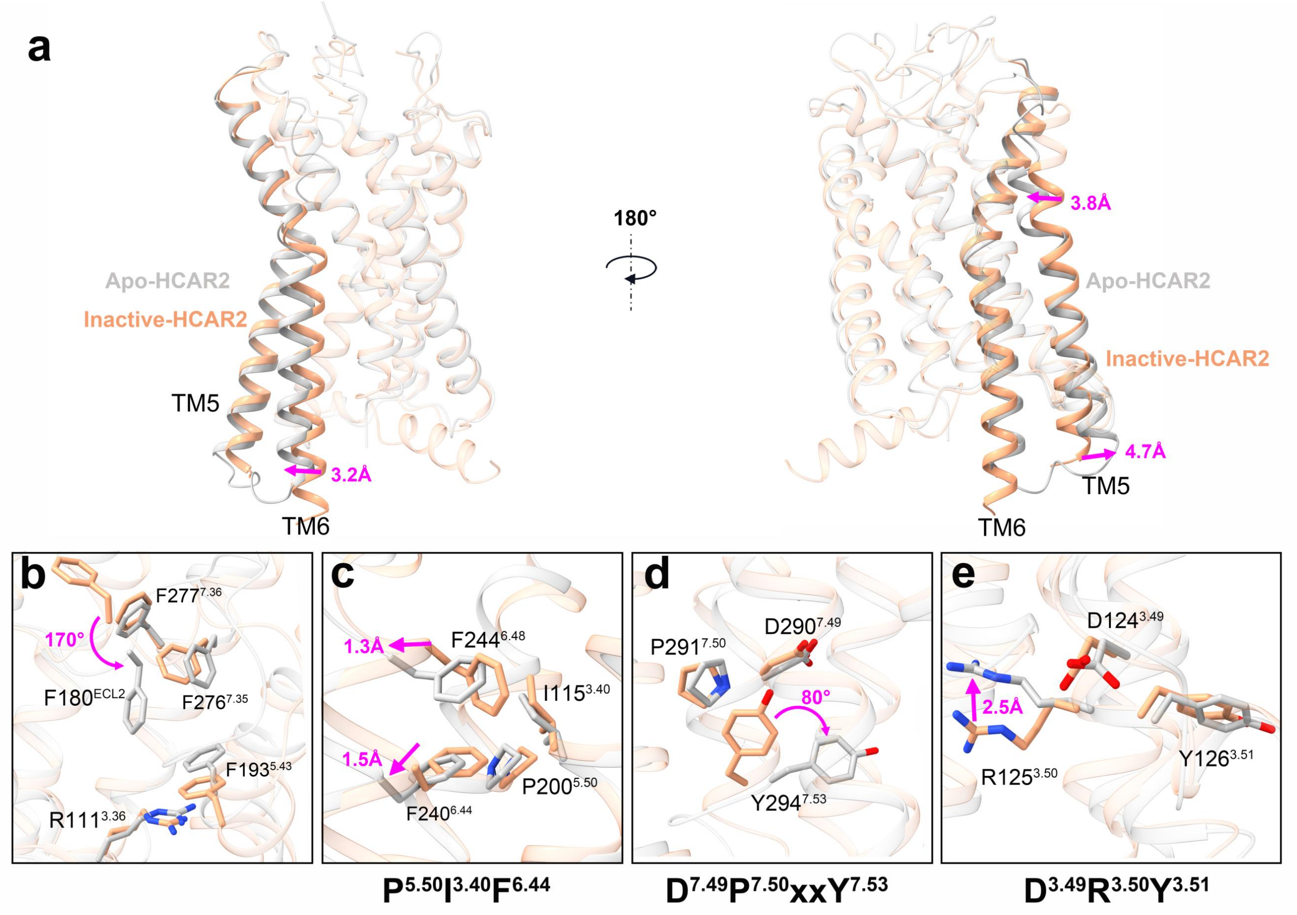

Supplementary Fig. S20 Comparison of the apo state of HCAR2 with the inactive HCAR2 (PDB: 7ZLY).

a Shifts of TM5 and TM6 in the apo state of HCAR2 relative to the inactive state. Superposition of the key residues (F180^{ECL2}, F276$^{7.35}$, F277$^{7.36}$, and F193$^{5.43}$) **(b)**, P$^{5.50}$-I$^{3.40}$-F$^{6.44}$ **(c)**, D$^{7.49}$P$^{7.50}$xxY$^{7.53}$ **(d)**, and D$^{3.49}$R$^{3.50}$Y$^{3.51}$ **(e)** motifs in the apo and inactive state of HCAR2. Light gray, apo-HCAR2; light orange, inactive HCAR2; magenta arrow, shift with respect to the inactive state.

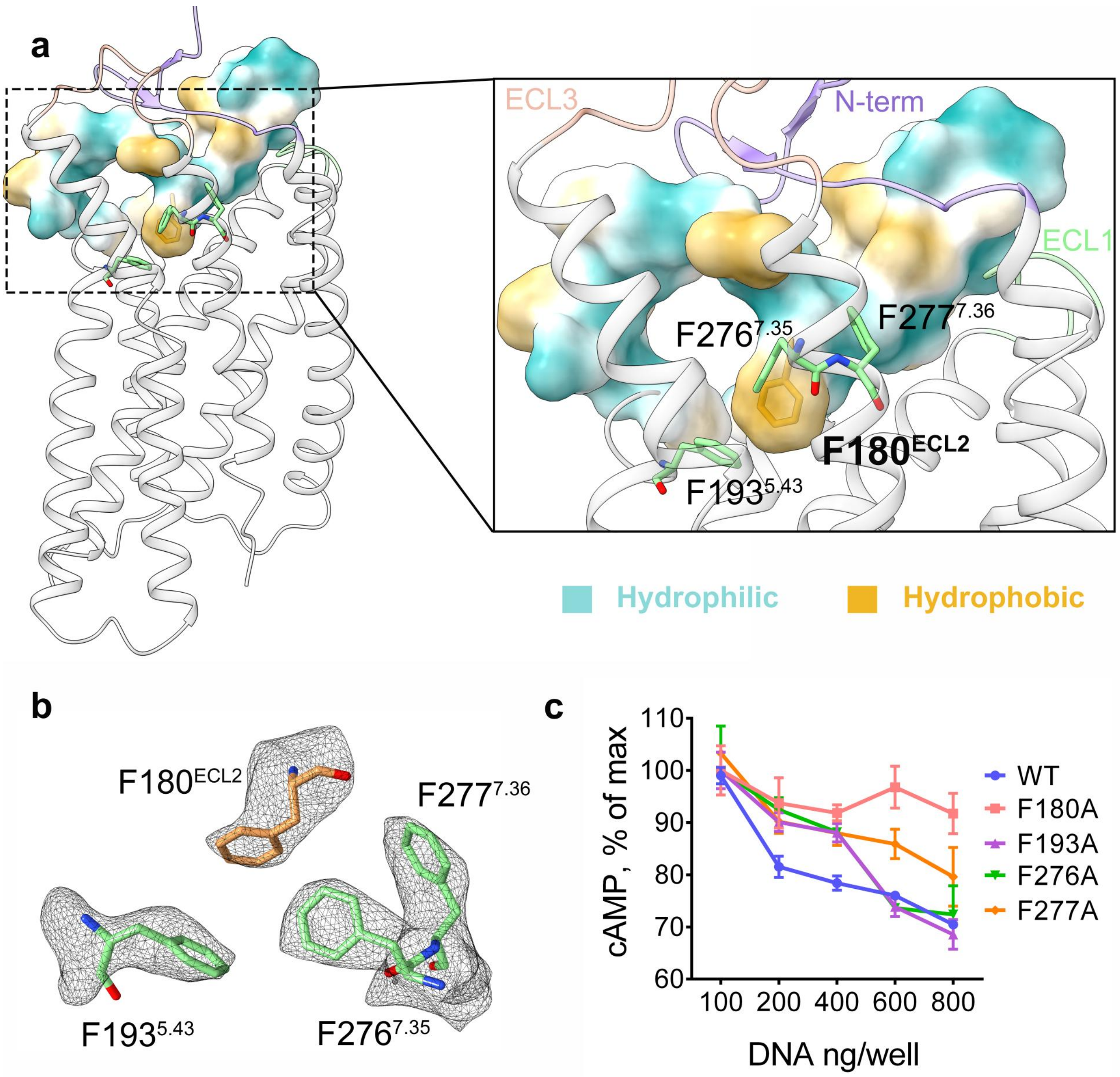

Supplementary Fig. S21 Structural features of HCAR2 in ECL2 region.

a Hydrophobic residue F180^{ECL2} (gold) in ECL2 deeply inserts into the orthosteric pocket. The hydrophilic and hydrophobic regions in ECL2 are shown as turquoise and gold surfaces, respectively. The hydrophobic residues F276$^{7.35}$, F277$^{7.36}$, and F193$^{5.43}$ in the orthosteric pocket are shown as lawn green sticks. **b** Density maps of key residues F180^{ECL2}, F276$^{7.35}$, F277$^{7.36}$, and F193$^{5.43}$. The density maps are shown at a contour level of 3.6 rmsd. **c** Effects on Gi-mediated cAMP by mutating the key residues F180^{ECL2}, F276$^{7.35}$, F277$^{7.36}$, and F193$^{5.43}$. The data are presented as mean ± SEM. The experiments were performed in triplicate.

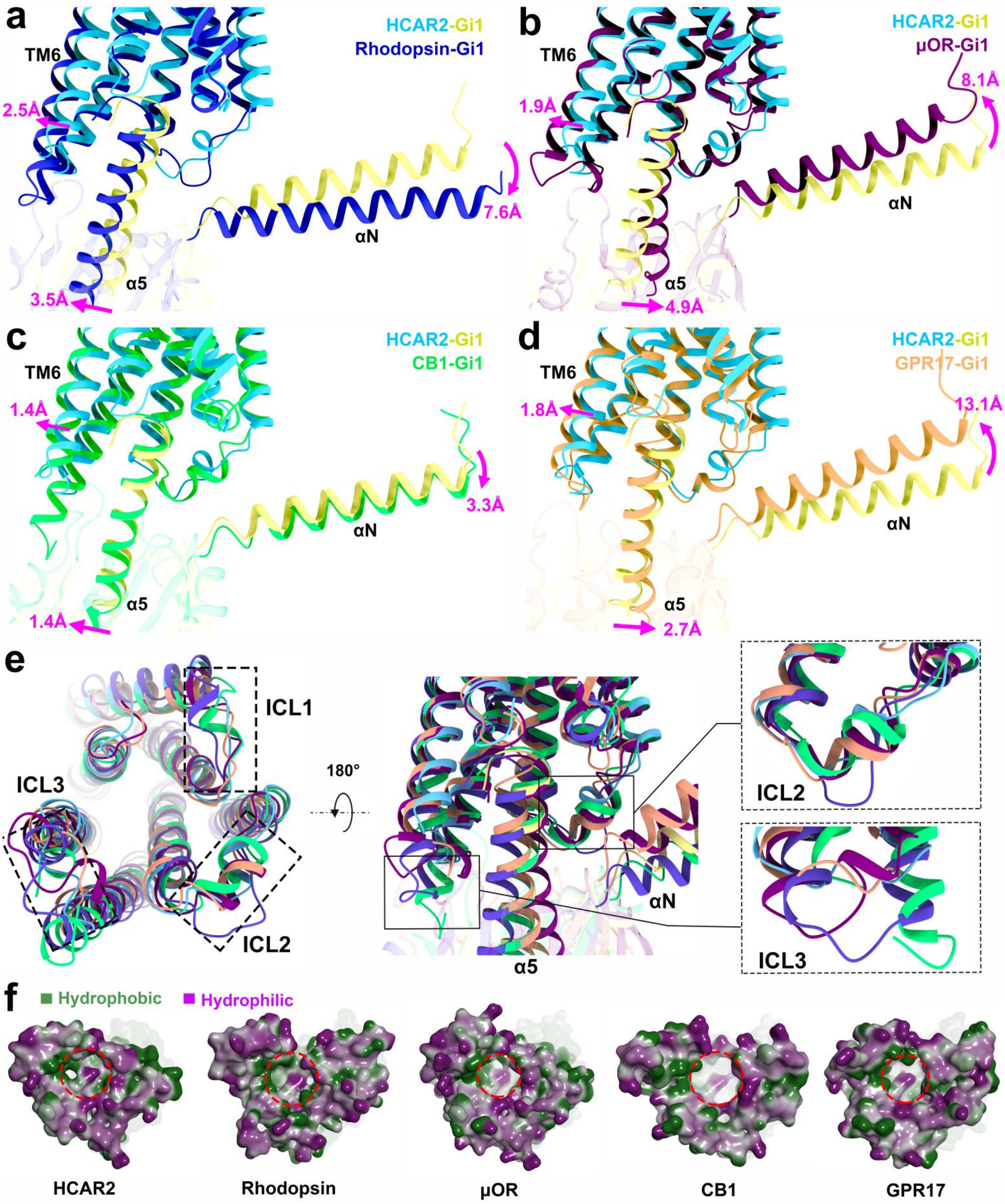

Supplementary Fig. S22 Pairwise comparisons of HCAR2-Gi1 versus rhodopsin-Gi1 (a), μOR-Gi1 (b), CB1-Gi1 (c), and GPR17-Gi1 (d). Magenta arrow indicates the shift with respect to HCAR2-Gi1. **e** Superimposition of the intracellular regions of HCAR2-Gi1 (deep sky blue and light yellow), rhodopsin-Gi1 (slate blue), μOR-Gi1 (dark magenta), CB1-Gi1 (turquoise), and GPR17-Gi1 (dark salmon). **f** G-protein binding pockets of HCAR2, rhodopsin, μOR, CB1, and GPR17. The pockets and ligands are shown as surfaces and colored according to hydrophobicity (green) and hydrophilicity (magenta).

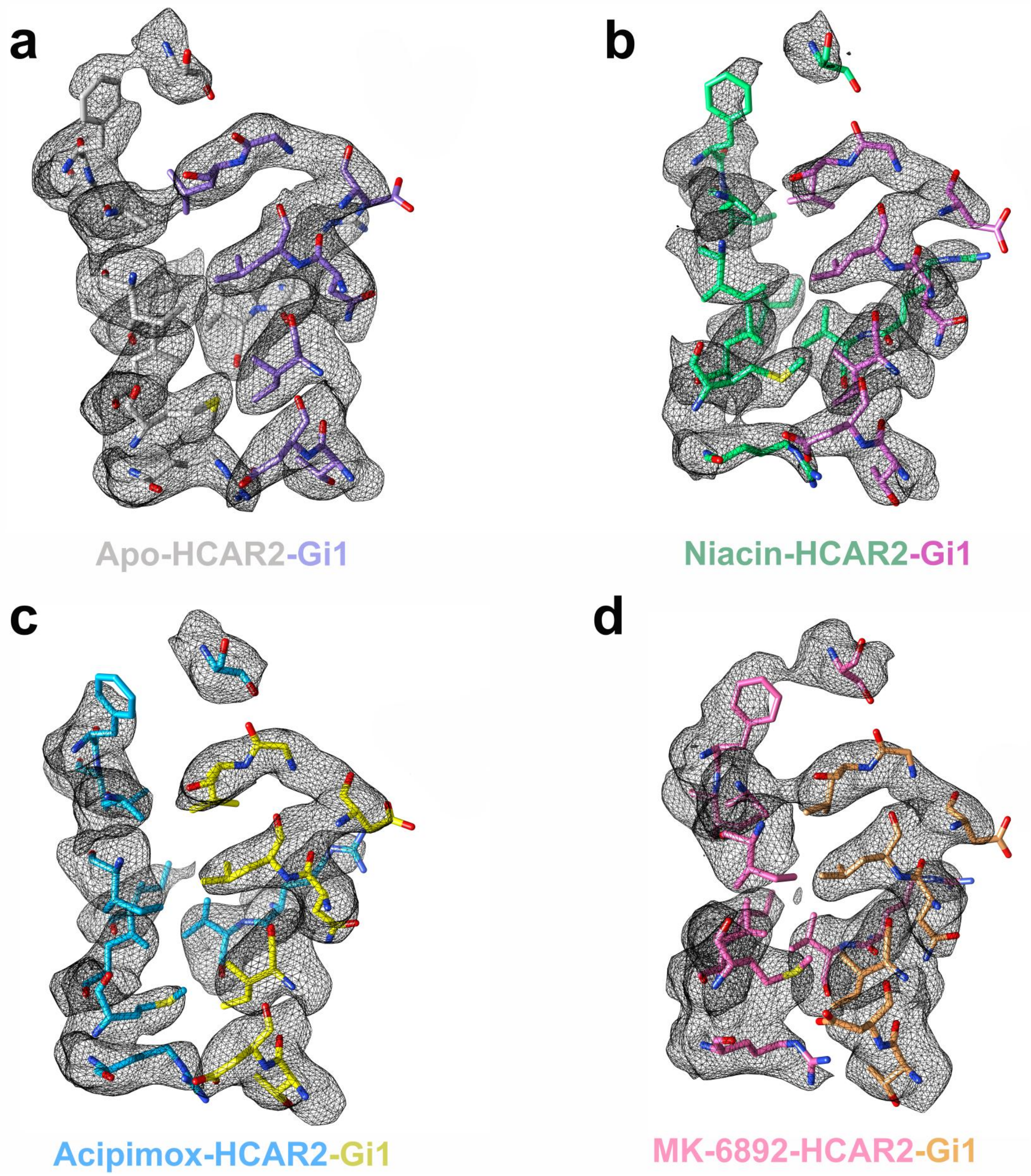

Supplementary Fig. S23 Density maps of the amino acids of the G protein interface in the apo (a), and niacin- (b), acipimox- (c), MK-6892-bound (d) HCAR2 complexes.

Light gray and purple, apo-HCAR2-Gi1; forest green and plum, niacin-HCAR2-Gi1; deep sky blue and light yellow, acipimox-HCAR2-Gi1; hot pink and orange, MK-6892-HCAR2-Gi1. The density maps are shown at a contour level of 3.6 rmsd.

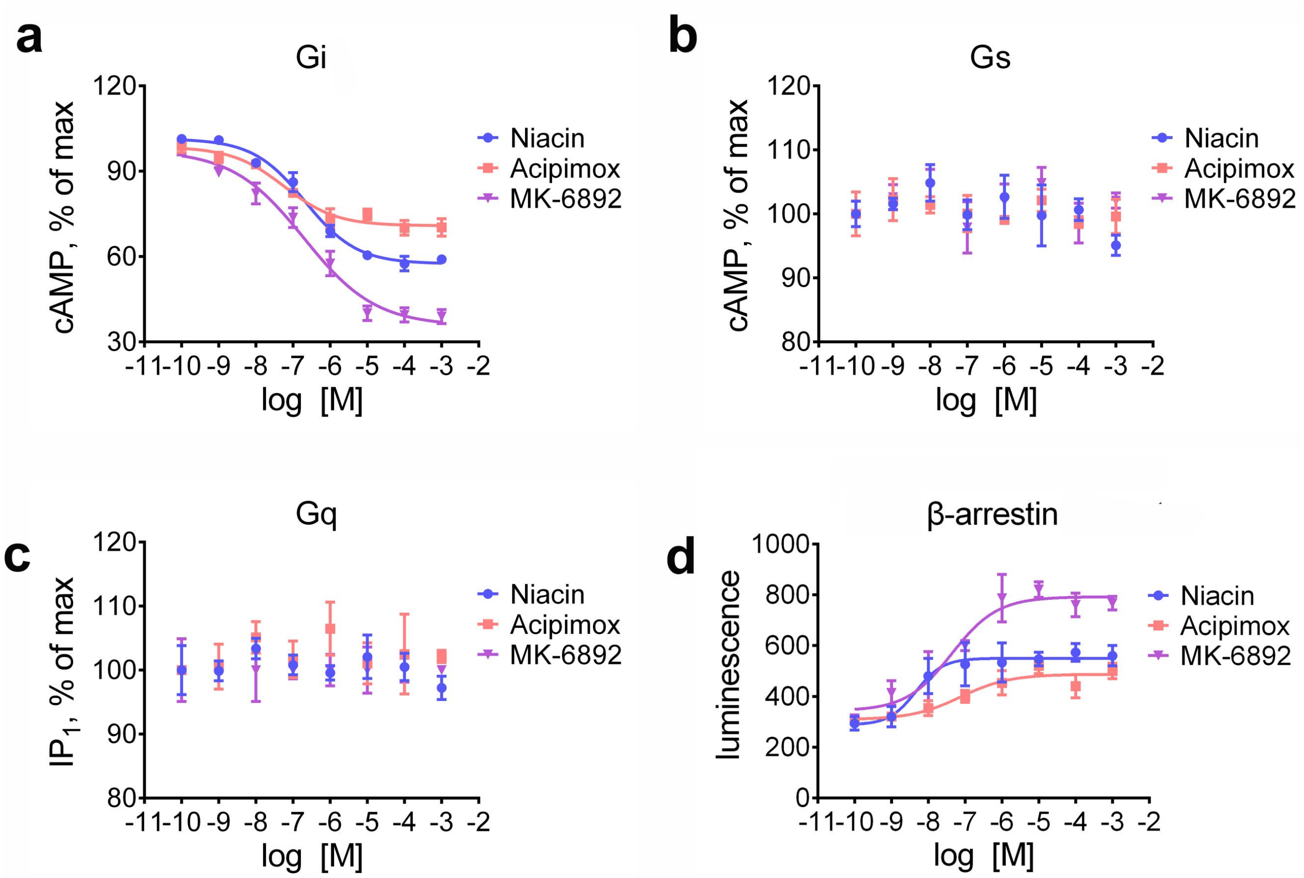

Supplementary Fig. S24 Coupling preference of HCAR2 to several G protein subtypes, including Gi (a), Gs (b), and Gq (c), as well as β-arrestin recruitment (d) in response to the activation mediated by niacin, acipimox, and MK-6892.

The data are presented as mean ± SEM. The experiments were performed in triplicate.

Supplementary Table S1. Cryo-EM data collection, refinement and validation statistics.

**Data collection and processing**	**HCAR2-apo EMD-35463 PDB ID 8IJ3**	**HCAR2-niacin EMD-35483 PDB ID 8IJA**	**HCAR2-acipimox EMD-35484 PDB ID 8IJB**	**HCAR2-MK-6892 EMD-35485 PDB ID 8IJD**
Magnification	105,000	105,000	105,000	105,000
Voltage (kV)	300	300	300	300
Electron exposure (e /Å)	48.7	55.26	52.94	52.94
Defocus range (pm)	-1.0 to -2.0	-1.0 to -2.0	-1.0 to -2.0	-1.0 to -2.0
Pixel size (Å)	0.85	0.83	0.85	0.83
Symmetry imposed	C1	C1	C1	C1
Initial particle images (no.)	2,947,103	3,191,801	1,328,380	1,988,362
Final particle images (no.)	311,666	879,036	221,940	291,441
Map resolution (Å)	3.28	2.69	3.23	3.25
FSC threshold	0.143	0.143	0.143	0.143
**Refinement**				
Initial model used (HCAR2, Alphafold)	Q8TDS4	Q8TDS4	Q8TDS4	Q8TDS4
Initial model used (Gi1+scFV16, PDB)	6OMM	6OMM	6OMM	6OMM
Map sharpening B-factor (Å^2)	-50	-50	-50	-50
**Model composition**				
Non-hydrogen atoms	8957	8914	8985	9002
Protein residues	1134	1136	1136	1136
**Average B factor (Å^2)**				
Protein	74.07	75.56	113.95	148.20
Ligand		98.61	122.85	147.01
**R.m.s. deviations**				
Bond lengths (Å)	0.005	0.006	0.004	0.005
Bond angles (°)	0.644	0.616	0.665	0.835
**Validation**				
MolProbity score	1.73	2.21	2.03	2.20
Clashscore	7.13	20.75	11.09	19.36
**Ramachandran plot**				
Favored (%)	94.81	95.08	91.43	94.20
Allowed (%)	5.19	4.83	8.30	5.80

Supplementary Table S2. Residues with sidechain deletion due to the lack of density.

Region	HCAR2-apo	HCAR2-niacin	HCAR2-acipimox	HCAR2-MK-6892
N-terminal	12, 15	12	10, 12, 14-16, 22-23	11, 12-17
TM1	24, 25, 27, 30, 36, 39-44, 46, 47, 50-52, 54, 55	43, 50, 55	24, 28, 38, 40, 42, 43, 55	22, 24, 25, 27, 28, 34, 36-40, 42, 43, 45, 47, 50, 52, 54, 55
ICL1	56, 57	57	57	56-58
TM2	60	60	60	60, 67, 74
ECL1	94	-	-	92, 94
TM3	97, 101	101	101	101
ICL2	138	-	-	-
TM4	164-166	142, 165	142, 166	164-166
ECL2	167, 169, 185	184	167	167, 171
TM5	-	-	185, 190	184, 192, 219
ICL3	-	222	-	221, 222
TM6	258-261		-	253, 257, 258, 260, 261
ECL3	264	-	267	263, 264, 265, 267
TM7	296	-	270	296
Gαi1	28, 43, 205, 207, 229, 232, 261, 272, 279, 280, 289	43, 51, 245, 328	43, 205, 232, 233, 275, 279, 280, 289, 298	193, 205, 270, 272, 273, 277, 279, 280, 289, 298
